# Elucidating the role of multivalency, shape, size and functional group density on antibacterial activity of diversified supramolecular nanostructures enabled by templated assembly†

**DOI:** 10.1039/d2mh01117d

**Published:** 2022-10-24

**Authors:** Amrita Sikder, Amanda K. Pearce, C. M. Santosh Kumar, Rachel K. O’Reilly

**Affiliations:** a School of Chemistry, University of Birmingham Birmingham B15 2TT UK r.oreilly@bham.ac.uk; b Institute of Microbiology and Infection, School of Biosciences, University of Birmingham Birmingham B15 2TT UK

## Abstract

With the increased prevalence of antibiotic-resistant infections, there is an urgent need to develop novel antibacterial materials. In addition, gaining a complete understanding of the structural features that impart activity toward target microorganisms is essential to enable materials optimisation. Here we have reported a rational design to fabricate antibacterial supramolecular nanoparticles with variable shape, size and cationic group density, by exploiting noncovalent interactions between a shape determining template amphiphile and a cationic amphiphile to introduce charge on the nanoparticle surface. We have shown that the monomeric cationic amphiphile alone showed poor antibacterial activity, whereas nanostructures formed by co-assembling the complementary units showed significantly enhanced antibacterial efficiency. Further, the systematic variation of several structural parameters such as shape, spacing between the cationic groups and size of these nanostructures allowed us to elicit the role of each parameter on the overall antibacterial properties. Finally, we investigated the origin of the differing antibacterial activity of these nanoparticles having different shape and size but with the same molecular composition, by comparing the thermodynamic parameters of their binding interactions with a bacterial membrane mimic.

## Abstract 2

Supramolecular cationic nanomaterials of diverse nanostructure prepared by templated assembly. Size, shape and cationic group density of the nanomaterials play an important role in antibacterial activity.

## Graphical abstract

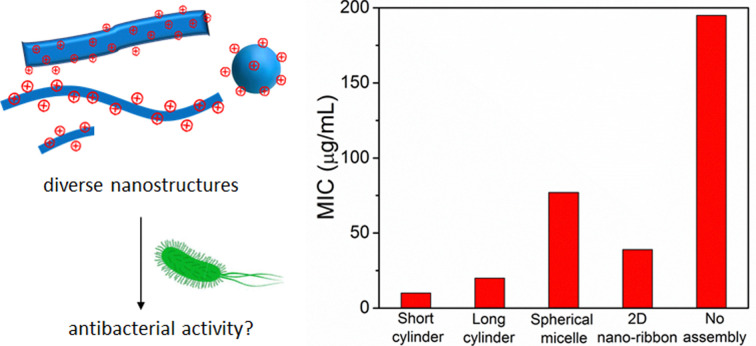

**New concepts**

Infectious diseases spread by antimicrobial resistant bacteria could potentially be the next pandemic spreading across multiple continents. Therefore, there is an urgent need to explore new antibacterial materials, and in particular to elucidate a thorough understanding of the structure property relationships of these materials to maximise their efficiency. Herein, we report a novel and versatile strategy to create antibacterial nanoparticles having different shape, size and functional group density, giving promising antibacterial properties. The nanoparticles have been prepared using noncovalent π–π interaction mediated assembly between electron rich and electron poor aromatic chromophore containing π-amphiphiles, where the different shapes were enabled by templated assembly. Unlike the existing literature, these tunable nanoparticles are generated from near identical building blocks having the same hydrophilic–hydrophobic balance (which is known to significantly affect antibacterial properties), making this system advantageous for unambiguously determining the structural features responsible for imparting activity toward the target microorganisms. Our data demonstrates that along with the multivalent effect, shape, size and proper placement of cationic functional groups all have a significant role to play to enable efficient interactions with bacterial membranes. The energy of interactions of these nanoparticles with a bacterial membrane mimic was quantitatively calculated to gain further insight into the origin of the very different antibacterial properties.

## Introduction

Infectious diseases caused by microbial infection remains one of the most serious problems in modern society.^1^ It has been found that almost half of the clinically reported infections are caused by *Escherichia coli* (*E. coli*) and *Staphylococcus aureus* (*S. aureus*).^2^ The continuous emergence of new antimicrobial resistant strains^3^ of these microorganisms against traditional antibiotics has led scientists to investigate new antibacterial therapies. Self-assembled cationic nanostructures including cationic peptides,^4–6^ small molecule surfactants^7–9^ and polymers^10–12^ have become the most appealing materials for demonstrating improved antimicrobial activity. The cationic head groups present on the surface of these self-assembled materials electrostatically bind to the negatively charged phospholipids present on the bacterial membrane, followed by insertion of the hydrophobic moiety initiating pore formation and subsequent disruption of the bacterial membrane eventually killing the microorganism.^13^ It has been reported that antibacterial activity can be controlled by several factors of the self-assembled nanomaterials, such as multivalency, charge density, structural rigidity, hydrophobicity, shape and size, therefore allowing researchers to test for improved antibacterial efficiency by tuning these parameters.^14–23^ For example, Sampson and co-workers tested the role of optimised distance between the cationic and hydrophobic groups and reported that evenly spaced functional groups lead to higher antibacterial efficiency.^18^ Jang and co-workers prepared a series of cationic polymer-modified silica spherical nanoparticles and found that small size particles showed better potency.^19^ Wang and co-workers demonstrated that peptide amphiphiles of varied spacer length can lead to different nanostructures which impacts the extent of antimicrobial activity.^21^

In this regard, Chen and co-workers prepared polymer nanoparticles of different shape but failed to observe any significant difference in their antibacterial performance.^22^ Overall, the literature to date is inconclusive as to how nanoparticle shape and size influence antibacterial activity. We suggest these very different observations could arise due to a lack of structural precision in the polymer structure owing to the high polydispersity, as the polymer chain length is also known to influence antibacterial properties.^24,25^ Further, the shape of polymeric nanoparticles is mostly directed by the packing parameter^26^*i.e.* the hydrophilic–hydrophobic balance, which is also an essential parameter for showing distinct antibacterial activity, thus limiting the ability to interpret the morphology and size effects exclusively. Similarly, in the case of small molecule amphiphiles^27^ and peptide amphiphiles,^21^ different building blocks have been employed to generate the varying shape nanostructures, making it difficult to correlate the relationship between these ordered structures and antibacterial activities. Therefore, one of the goals and ongoing challenges is to prepare diverse nanostructures without compromising the structural parameters of the molecular/macromolecular building blocks, in order to unambiguously understand which nanoparticle structural features that impart activity toward the target microorganism, facilitating the establishment of more efficient antibacterial materials.

Supramolecular polymers^28,29^ prepared *via* noncovalent interactions have been considered as a promising material due to their structural diversity and processability by reversible assembly–disassembly. The chemical structure of the supramolecular building block directs how the multiple noncovalent interactions will compete with each other, leading to nanostructures with stable conformations. Thus, it is possible to generate a diverse range of supramolecular nanomaterials by subtly tuning the noncovalent interactions present in the assembled system.^30–32^ Considering the structural diversity along with conformational and functional integrity, supramolecular polymers are an ideal candidate to form different nanostructures with high structural precision to screen for antibacterial activity.

**Scheme 1:**
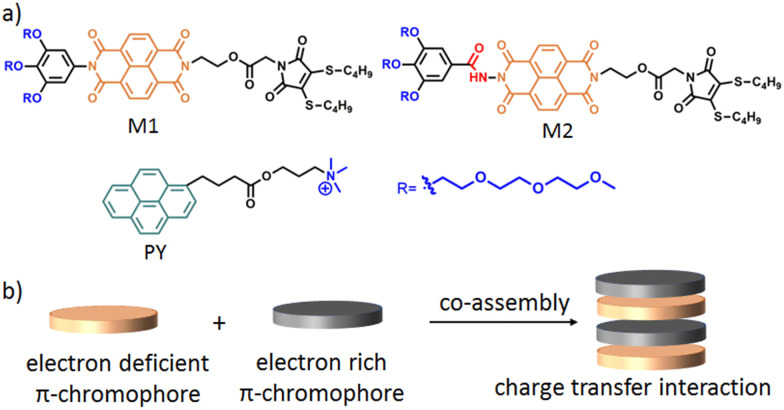
(a) Structure of M1, M2 and PY. (b) Schematic representation of π–π interaction mediated charge transfer complex formation.

In this context, we have recently reported^33^ the formation of different shaped nanoparticles (spherical micelles, cylindrical micelles and 2D-nanoribbons) by interplaying the H-bonding, hydrophobic and π–π interactions using structurally near-identical π-amphiphiles (M1 and M2 in Scheme 1) having the same hydrophilic–hydrophobic balance. Further to this, it is well established that charge-transfer (CT) interactions^34,35^ between electron deficient and electron rich chromophores can be utilised for the construction of supramolecular nanomaterials (Scheme 1b). We envisioned that co-assembling an electron deficient naphthalene-diamide (NDI) chromophore containing π-amphiphiles (M1 and M2) with a suitably functionalised electron rich chromophore *via* CT-interactions would lead to diverse nanostructures with functional features. Here, we have selected a quaternary ammonium functionalised pyrene (PY in Scheme 1a) to impart multiple positive charges on the co-assembled nanoparticle surface for antibacterial inhibition.

In this work, we demonstrate this unique strategy to prepare a set of nanoparticles having different shape, size, and cationic group density, while maintaining higher order structural precision. This enables us to investigate the effect of each variable on antibacterial activity in a more coherent manner compared to previously reported antibacterial materials. The salient features of these systems are: (i) high structural precision with comparable hydrophobic–hydrophilic balance to exclude the possibility of interference from other parameters on antibacterial activity; (ii) ease of synthesis: charge density is readily tunable by changing the ratio of the amphiphiles; (iii) facile access to different particle sizes without compromising the chemical composition/functionality of the nanoparticles due to the reversible nature of noncovalent supramolecular interactions.

## Results and discussion

### Synthesis and molecular characterisation

Neutral amphiphiles M1 and M2 were synthesised according to a previously reported procedure.^33^ Cationic amphiphile PY was synthesised following the synthetic scheme described in the ESI† (Scheme S1) and characterised by ^1^H-NMR (Fig. S1, ESI†), ^13^C-NMR (Fig. S2, ESI†) and ESI-MS spectroscopy. The co-assembly was investigated in aqueous medium unless otherwise specified. The detailed sample preparation procedures are described in the ESI.†

### Co-assembly and nanostructure characterisation: variable shape

**Fig. 1:**
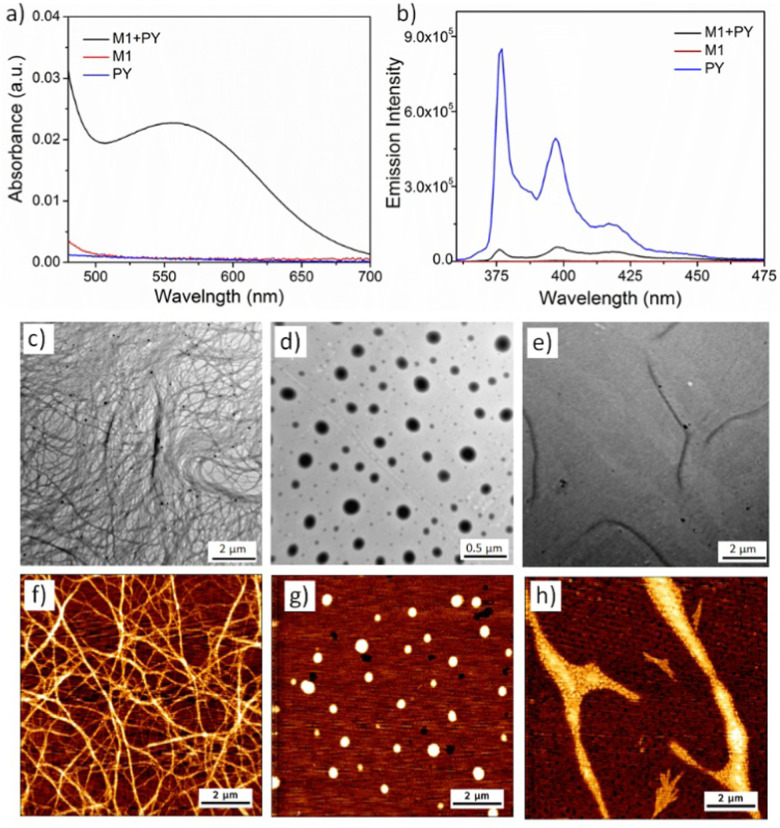
(a) UV-vis and (b) fluorescence spectra of aqueous solution of 1 : 1 mixture of M1 + PY, PY and M1. TEM images of 1 : 1 co-assembly of M1 + PY (c), M2 + PY fresh solution (d) and M2 + PY aged solution (e). AFM images of M1 + PY (f), M2 + PY fresh solution (g) and M2 + PY aged solution (h). Concentration of the sample – 0.5 mM for UV-vis fluorescence and TEM. Concentration used for AFM is 0. 25 mM. Cuvette path length – 0.1 cm, slit used for fluorescence −1.

In order to probe the solution properties of PY, solvent dependent UV-vis and fluorescence spectra were measured (Fig. S3, ESI†). Sharp peaks at 343 nm, 325 nm and 312 nm in tetrahydrofuran (THF) solution indicated the absence of π–π stacking between the pyrene chromophores and the existence of non-assembled monomeric PY.^36^ Very similar absorption spectra were recorded for an aqueous solution of PY, confirming that PY does not form any self-assembly and prefers to remain as monomers in aqueous medium as well. The absence of any excimer^37^ formation in the fluorescence spectra also indicated the lack of any π–π interaction, further suggesting PY doesn’t self-assemble. This is likely due to electrostatic repulsion among the cationic head groups along with electronic repulsion between the electron rich pyrene chromophores. On the other hand, amphiphile M1 is known to form micrometre long cylindrical micelles *via* π–π stacking and hydrophobic interactions.^33^ When M1 and PY were co-assembled (detailed sample preparation has been described in ESI†) in 1 : 1 ratio (calculated in mole %), UV-vis of the aqueous solution showed the emergence of a new peak at 550 nm (Fig. 1a) indicating the formation of the CT-complex by alternate stacking of the M1 and PY chromophores.^38^ Quenching of the PY fluorescence^39^ (Fig. 1b) in the presence of M1, further confirmed intercalation of the pyrene chromophore into the NDI-chromophoric stack. The association constant between M1 and PY was found to be 3.8 × 10^4^ M^−1^ as determined by concentration dependent UV-vis spectra (Fig. S4, detailed procedure has been described in ESI†).^40^ Dry state TEM (Fig. 1c) and AFM (Fig. 1f) images of the co-assembly confirmed formation of the micrometre long cylindrical micelles having a width of 7 ± 0.5 nm. Finally, a positive surface charge on the cylinder surface due to the PY units was confirmed by zeta potential (+29 mV) measurements.

Similarly, we investigated the co-assembly properties of a 1 : 1 mixture of PY and M2 amphiphiles in aqueous solution. As above, the appearance of the CT band in UV-vis spectroscopy (Fig. S5a, ESI†) and quenching of the PY fluorescence (Fig. S5b, ESI†) confirmed co-assembly *via* donor–acceptor alternate chromophore stacking. HRTEM (Fig. 1d) and AFM (Fig. 1g) images confirmed the formation of spherical micelles. The diameter of the micelle was calculated to be 200 ± 50 nm by imageJ analysis, with dynamic light scattering (DLS) measurements providing a similar size (Fig. S6, ESI†). A positive zeta potential (+20 mV) confirmed the cationic surface charge of the micelles. We have previously observed that M2 alone can self-assemble into spherical nanoparticles, however, transform into more stable nanoribbon structures by chromophoric reorganisation^41^ within 12–14 days.^33^ To check the possibility of such transformation with the M2 + PY co-assembled system, we monitored the time dependent morphology. Pleasingly, time dependent AFM images revealed a gradual formation to 2D-nanoribbons (Fig. 1h and Fig. S7, ESI†). Notably, the time taken for the full transformation of the M2 : PY co-assembled system is much longer (28–30 days), which can be attributed to slower reorganisation and recombination due to repulsive interactions among the cationic nanoparticles. Height measurement (Fig. S8, ESI†) of the AFM image displayed a height of ∼7.6 nm and 0.5–0.7 nm width of the nanoribbons. HRTEM images (Fig. 1e) further confirmed the formation of the 2D-nanoribbons. UV-vis spectra (Fig. S9a, ESI†) and fluorescence spectra (Fig. S9b, ESI†) of the aged solution indicated intact CT-interactions in the nanoribbon structures. No significant differences in the association constant between PY and M2 were observed for the two nanostructures (6.3 × 10^3^ M^−1^ and 7.0 × 10^3^ M^−1^ respectively for spherical micelle and nanoribbon; Fig. S10 and S11, ESI†). However, the zeta potential slightly increased to +28 mV which we attribute to the change in the surface area of the nanoparticles during the zero-dimensional micelle to two-dimensional nanoribbon transformation. Further, time dependent spectroscopy (Fig. S12, ESI†), DLS (Fig. S13a and b, ESI†), zeta potential measurement (Fig. S13c and d, ESI†) and microscopy study (Fig. S14, ESI†) confirmed good stability of the cationic nanoparticles in aqueous medium.

### Nanostructure preparation with variable charge density

In order to prepare a series of nanoparticles with a different surface charge density, the amount of PY was varied in the two component co-assembled systems. It was found that cylindrical micelles formed at different ratios of M1 : PY were stable until 50% PY incorporation (1 : 1 ratio), beyond this the particles collapsed and generated undefined shapes (Fig. S15, ESI†). Disassembly of the cylindrical nanoparticles at a higher percentage of PY was further confirmed by the appearance of smaller peaks in DLS measurements (Fig. S16, ESI†). It is anticipated that this inability to assemble beyond the 1 : 1 ratio is due to the higher extent of electrostatic repulsion and lack of interaction between the pyrene chromophore itself. As expected, an increase in positive zeta potential was observed with increasing PY ratio in the co-assembled system, confirming the enhancement of surface charge density with increase in the amount of PY (Fig. S17, ESI†). In a similar manner, spherical micelles (Fig. S18, ESI†) and 2D-nanoribbons (Fig. S19, ESI†) with different surface charge densities were prepared by varying the PY amount from 0% to 50% percent into the M2 assembly. Zeta potentials were measured to confirm a gradual increase in charge density (Fig. S17, ESI†) on the spherical micelles and nanoribbons with increasing amount of PY intercalation.

### Molecular packing in different nanostructures

**Scheme 2:**
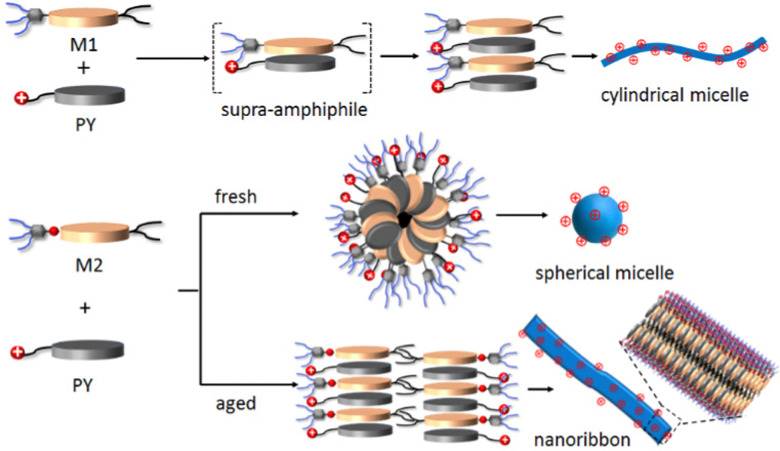
Schematic representation of proposed molecular packing of M1, M2 and PY in different nanostructures.

All the microscopic and macroscopic studies revealed that the nanostructure shape can be modulated by subtly tuning the noncovalent forces presence in the amphiphiles. We have reported earlier^33^ that amphiphile M1 self-assembles into cylindrical micelles *via* π–π stacking, whereas the presence of H-bonding in the case of M2 changes the morphology to spherical micelles (kinetically trapped state)^42^ and finally towards more thermodynamically favourable 2D-nanoribbons. Here, we have shown that this structural information associated with M1 and M2 amphiphiles could be further translated into the M1 + PY and M2 + PY binary systems to direct the co-assembly, where M1/M2 act as the shape determining template for obtaining the desired shape of the cationic nanoparticles.^30,43^ A proposed model of molecular packing to rationalise the observed microscopic images and spectroscopic results is depicted in Scheme 2. M1 and PY amphiphiles initially form heteroatomic dimers (known as supra-amphiphile)^44^*via* CT-interaction, and then hierarchically assemble to generate cylindrical micelles. Here, the core consists of hydrophobic alkane chains and the hydrophilic oligo-oxy chains of M1 and positively charged quaternary amine groups of PY remain on the shell generating the cationic surface. M2 and PY form similar supra-amphiphile *via* CT-interactions along with H-bonding (H-bonding between M2 and PY was probed by IR; Fig. S20 and S21a, ESI†) to generate spherical micelles with a cationic surface, which further reorganise into a bilayer orientation to form cationic nanoribbons. It should be noted that H-bonding interactions restrict the complete overlap of the donor and acceptor chromophores in the M2-PY complex (Fig. S21a, ESI†), leading to a decrease in the association constant compared to the M1-PY complex having complete chromophoric overlap due to an absence of such constraints (Fig. S21b, ESI†).^45^

### Antibacterial activity evaluation: effect of shape and charge density

**Fig. 2:**
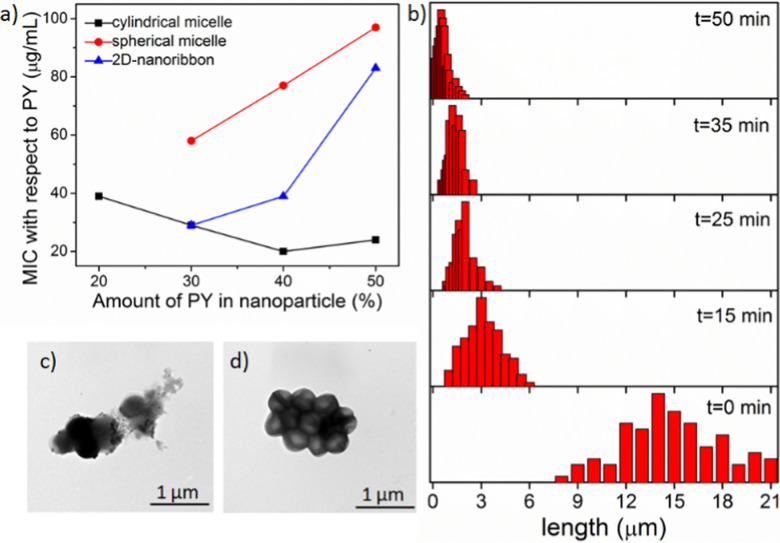
(a) Comparison of MIC values (*S. aureus*) with different nanoparticles having different charge densities; (b) histogram plot of size of cylindrical nanoparticles obtained by adjusting sonication time; (c) TEM image of lysed cells with distorted membranes upon treatment with ∼4 μm cylindrical micelles and (d) untreated healthy *S. aureus* cells.

To quantify the antibacterial activity, the killing efficacy was calculated quantitatively by determining the minimum inhibition concentration (MIC) value, *i.e.* the minimum concentration of the nanoparticle needed for complete inhibition of bacterial growth. We screened all the nanoparticles of different shape and charge densities (up to 50% PY content as discussed above) to evaluate the antibacterial activity (Table S1 in ESI†) against two different bacteria strains: Gram-positive *S. aureus* and Gram-negative *E. coli*. From the MIC data, it is evident that these nanoparticles exerted efficient antibacterial activity towards Gram-positive *S. aureus*, however were not very effective on Gram negative-*E. coli.* This was likely due to the presence of the protective double-layer membrane making it more challenging for the nanoparticle to interact with the Gram-negative bacteria.^46,47^ To determine the optimal distance between the PY to exert highest antibacterial activity, we have calculated the MIC values in terms of the amount of PY present in each nanoparticle (Fig. 2a, Table S2 and S3 in ESI†). Analysis of MIC values indicated that nanoparticles with 30–40% PY content were more effective than the nanoparticles containing 50% PY (Fig. 2a) indicating the necessity of an optimal distance (>10 Å)^18,48,49^ between the cationic groups in order to exhibit higher antibacterial efficiency. Interestingly, cylindrical nanoparticles showed the lowest MIC (20 μg mL^−1^) whereas the spherical nanoparticles showed the highest MIC (58 μg mL^−1^), with the nanoribbons exhibiting an intermediate activity (29 μg mL^−1^). From these data it is evident that the shape and the charge density of nanoparticles clearly play a role in the antibacterial activity. On the other hand, the antibacterial activity of the free PY (non-assembled monomeric solution) showed an even higher MIC (195 μg mL^−1^) indicating lowest antibacterial efficacy. This observation indicates that the multiple arrays of appended cationic groups displayed in a confined molecular arrangement cause locally increased concentrations of surficial cationic units, which significantly enhance interactions with the bacterial membrane compared to the discrete cations present in free PY solution. Overall, these results demonstrate the importance of the multivalent effect on antibacterial activity.^50,51^

### Effect of nanoparticle size

Since the cylindrical micelles (M1 : PY at 3 : 2 ratio) exhibited the highest antibacterial activity among all the nanoparticles, we were keen to next explore the effect of cylinder length. As these cylinders are formed through relatively weak noncovalent bonds, they can be broken into smaller cylinders using external forces, such as ultrasonication.^52,53^ Here, we generated the desired length cylinders by adjusting the sonication time (Fig. 2b, and Fig. S22, detail sample procedure has been described in ESI†). MIC values calculated with these varied length cylinders revealed a higher killing efficiency for shorter (3–4 μm) cylinders with a two times lower MIC (10 μg mL^−1^, Fig. 2a) value compared to the long (>10 μm) cylinders (MIC −20 μg mL^−1^). However, upon further truncation, the MIC value did not alter (Fig. S23, ESI†) confirming a threshold length of nanoparticles for efficient interactions with bacterial membrane of ∼4 μm.^20^

As the nanoparticles showed distinct growth inhibition propensities in the liquid cultures, we next assessed their antibacterial activity on solid surface. For this aim, we used a zone clearance assay^54,55^ to test the ability of the nanoparticles to inhibit *S. aureus* growth (Fig. S24, ESI†), wherein a clearance in the bacterial lawn represents growth inhibition and the cleared zone size represents the inhibition efficiency. Here, similar to the MIC values, the cylindrical nanoparticles exhibited the highest antibacterial activity even in the solid media, demonstrating their versatility in potential applications.

### Antibacterial activity mechanism

**Fig. 3:**
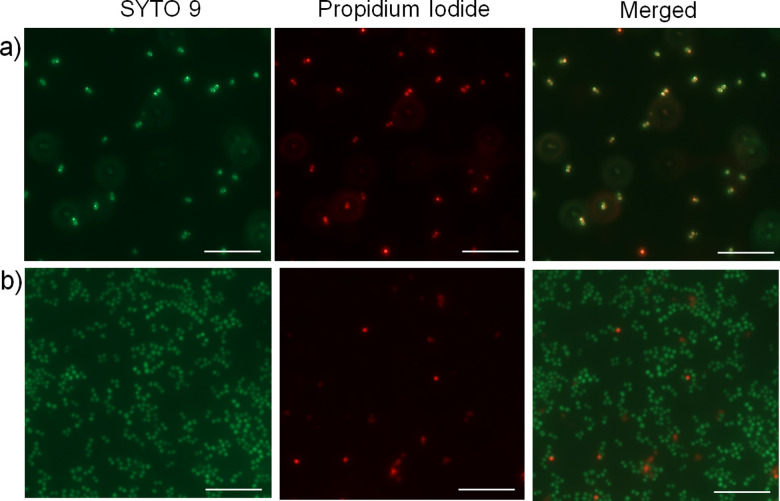
Fluorescence micrographs of *S. aureus* treated (a) with and (b) without short cylindrical micelles, followed by labelling with the indicated fluorescent probes. Images showing the propidium iodide staining depict the dead bacterial cells. Scale bar is 10 μm.

Next, we wanted to investigate whether the activity of the nanoparticles is due to membrane interaction and disruption. To test this, we employed a fluorescence-based bacterial live/dead assay, which employs two DNA intercalating fluorescent dyes; a green emitting fluorescent dye (SYTO 9) that can pass through bacterial membranes and stain the DNA of live or dead cells, and a red emitting dye (propidium iodide) that can permeate only through the damaged membranes and thus stain only the dead cells.^56^ Fluorescence microscopy images depict a significantly higher number of non-viable *S. aureus* cells upon treatment with the cationic cylindrical nanoparticles (Fig. 3a) compared with the untreated cells (Fig. 3b), demonstrating that treatment with the cylindrical nanoparticles significantly reduced *S. aureus* viability and membrane integrity, possibly through membrane disruption. Notably, owing to the bactericidal activity of the cylindrical nanoparticles, they slowed the cell growth and resulted in significantly lower cell density than the untreated cultures. Further, to examine whether these nanoparticles affect the cellular morphology, we employed TEM to image the *S. aureus* cells upon treatment with sub-MIC levels of the cylindrical nanoparticles. The TEM images revealed distinct morphological differences in the treated samples. While the untreated bacteria exhibited the characteristic smooth and coccus cellular surface, the bacteria treated with cylindrical nanoparticles exhibited lysis and significantly distorted membranes (Fig. 2c, d and Fig. S25, ESI†).^57^

### Origin of different antibacterial activity

The bacteria cell exhibits a negatively charged surface at physiological pH owing to the presence of various phosphoryl groups on the cell wall.^58–60^ As a result, positively charged nanoparticles can electrostatically bind on the surface of the bacteria leading to the disruption of the bacterial membrane causing cell death. Therefore, the extent of interaction highly depends on the contact area (which depends on the size and shape) of the nanoparticle surface with bacteria membrane. In an attempt to understand the origin of distinct antibacterial activity of these nanoparticles having different shape and sizes, we quantitatively calculated the thermodynamics of interaction of the nanoparticles with model lipid membranes using isothermal titration calorimetry (ITC).^61^

**Fig. 4:**
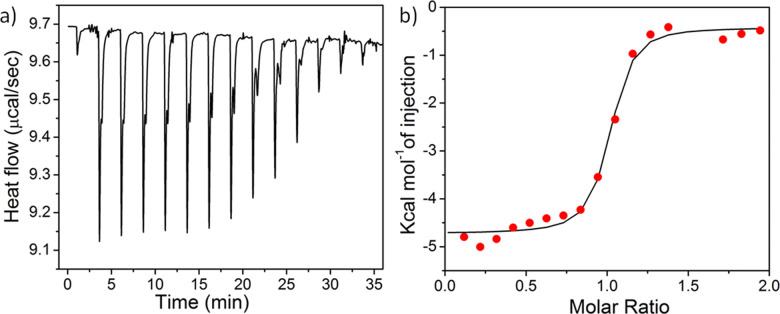
(a) Raw data of the isothermal titration of long cylinders with POPE:POPG liposome at 25 °C and (b) corresponding enthalpogram measured by peak integration as a function of the lipid/nanoparticle molar ratio.

Bacteria cell membrane mimicking liposomes were prepared using a 3 : 1 ratio of 1-palmitoyl-2-oleoyl-*sn-glycero*-3-phospho-(1′-rac-glycerol) (POPG) and 1-palmitoyl-2-oleoyl-*sn-glycero*-3-phosphoethanolamine (POPE), whereas 1-palmitoyl-2-oleoyl-*sn-glycero*-3-phosphoethanolamine (POPC) vesicles were prepared to mimic mammalian cell membranes.^62^ POPG/POPE (3 : 1) liposomes were injected into a solution of different nanoparticles and a significant heat change was noticed followed by saturation (Fig. 4 and Fig. S26–28, ESI†) for all the nanoparticles, indicating prominent interactions of the nanoparticles with the bacterial cell mimicking membrane. The binding constants (Ka) between the nanoparticles and lipid membranes, as well as the energy of interactions including Gibbs free energy (Δ*G*), change in entropy (Δ*S*), and change in enthalpy (Δ*H*) upon interaction were evaluated by fitting the raw data using the ‘one set of site’ model (Table 1). The trend of Ka values follow the order: spherical micelle < 2D-nanoribbons < long cylinders < short cylinders, indicating the highest binding affinity for short cylinders whereas the spherical micelles exhibited lowest binding affinity for the liposomes. Lowest enthalpy of interaction (Δ*H*) in case of spherical micelles further confirmed least electrostatic attraction between the nanoparticle and the bacteria attributed to smaller and less adaptive spherical nanostructures compared to open end micrometer long 1D-cylindrical or 2D-nanoribbons.^31^ In addition, another reason could be the unavailability of all the cationic functional groups due to kinetic entrapment of some of the PY inside the core of the nanostructures. Kinetic trapping can be supported by the lower value of zeta potential obtained for 1 : 1 co-assembly of M2 + PY into spherical micelles (+20 mV) compared to M2 + PY nanoribbons (+28 mV) as mentioned earlier (Fig. S17, ESI†). On the other hand, both cylindrical micelle and 2D-nanoribbon have micrometre long length, however the width of the cylindrical micelle is much smaller than the 2D-nanoribbon, which makes a significant difference in effective contact area between nanoparticle and bacteria membrane. Smaller width of the cylindrical nanoparticles makes it more flexible than the 2D-nanoribbon possessing higher contact area between the particles and the membrane by optimum local curvature of the particle at the contact point^63^ leading to greater extent of disruption of the bacterial membrane. This was further supported by the Gibbs free energy of interaction (Δ*G*) between the nanoparticle and bacterial membrane lipid membrane, which follow the order-spherical micelle < 2D-nanoribbons < cylindrical micelle.

**Table 1:** Energy of interactions of different nanoparticles with a bacterial membrane mimic as obtained by ITC

Nanoparticle	Ka (× 10^−3^ M^−1^)	Δ*G* (kcal M^−1^)	Δ*H* (kcal M^−1^)	Δ*S* (Cal M^−1^)
Long cylinder	2.6 ± 0.1	−9.2	−4.7 ± 0.07	15.6
Short cylinder	1.4 ± 0.03	−8.8	−4.7 ± 0.11	14.0
Nanoribbon	0.8 ± 0.1	−8.3	−4.8 ± 0.08	12.2
Sphere	0.4 ± 0.1	−6.3	−2.6 ± 0.06	9.6

Further, the positive Δ*S* values produced by the interaction can be attributed to the increase in entropy arising due to liposome membrane disruption, desolvation of the nanoparticles as well as desolvation of the lipid membranes as a consequence of nanoparticle–liposome interaction.^64^ Δ*S* values follow the order of: spherical micelle < nanoribbon < cylindrical micelle indicating that cylindrical micelles were able to penetrate through the lipid membrane more efficiently probably due to the presence of sharp edges onto the particle.^65^ However, it is impossible to get a straightforward relation between the size of dissimilar morphology on antibacterial activity, as several parameters such as surface area, flexibility as well as presence of sharp edges in the nanostructure can contribute to the final antibacterial activity. To have unambiguous interpretation of the size effect, we have prepared cylindrical micelles of different length and found that shorter cylinders exhibited a higher extent of interaction (Ka, Δ*H*, Δ*S*, Δ*G*) owing to a higher degree of flexibility and mobility than the longer one. Additionally, the presence of higher number of sharp edges enhances the ability of short cylinders to insert into and disintegrate the bacterial cell membrane leading to a higher extent of bacteria killing.

On the other hand, mammalian cell membranes are composed of zwitterionic lipids and cholesterol making the overall surface charge neutral, hence they do not exert any electrostatic binding with the cationic nanoparticle. Lack of any interaction was proved by no significant change in enthalpy in the ITC experiment performed with the mammalian lipid membrane mimic (POPC liposomes) (Fig. S29, ESI†), confirming the high selectivity of these nanoparticles toward bacterial cells over mammalian cells. In fact, an ideal antibacterial material should exhibit a selective toxicity against bacterial cells over mammalian cells. Red blood cells (RBCs) are commonly employed to assess such selectivity,^66^ wherein haemolysis of the RBCs in the presence of surface-active components is assessed using blood agar plates. Horse blood agar plates were used to test the ability of the nanoparticles to lyse RBCs (Fig. S30, ESI†).^67^ From the experiment, it is evident that the nanoparticles did not bring about haemolysis even at 4 mM (1 : 1 ratio) stock concentrations (760 μg mL^−1^ of PY), which is much higher than their respective MICs. Whereas a positive control experiment performed with sodium dodecyl sulphate (SDS) showed haemolysis (as evident from white zone formation) of the RBC.^68^ This observation demonstrated that the nanoparticles are non-toxic antimicrobial agents and indicated their potential antimicrobial applications.

## Conclusions

In conclusion, we have rationally designed and synthesised a series of cationic nanoparticles of different shape, size and functional group density using two-component supramolecular assembly to evaluate their antibacterial properties. The distinct packing mode of M1 + PY and M2 + PY led to different assemblies forming distinctly different nanoparticles. By changing the ratio of M1/M2 : PY we could achieve nanoparticles with a desired surface charge. We have demonstrated that the formation of nanoparticles strongly influences the antibacterial efficacy due to the multivalent effect. Comparison of the MIC values of these nanoparticles on two different strains of bacteria revealed a significant effect of the nanoparticle shape and size on antibacterial activity. Small cylindrical micelles exhibited the highest antibacterial activity (10 μg mL^−1^) over the long cylindrical micelles, 2D-nanoribbons and spherical micelles. Further, we have demonstrated that the optimal spacing of the cationic groups play an important role for efficient interaction with the bacterial cell membrane. Finally, the origin of the different antibacterial activities was evaluated by probing the energy of interactions of these nanoparticles with bacterial cell membrane mimics. Through energy of binding calculations, it was found that both enthalpy and entropy contributed towards favourable binding, where the highest entropic contribution for short cylinders was attributed to fast rupture of the bacterial membrane. Overall, these nanoparticles showed promising antibacterial activity and good selectivity towards mammalian cells. We envision that the design principle of these nanostructures and the findings regarding structure–property relationships of these nanostructures provides guidance for making increasingly active and potentially therapeutic functional materials for antibacterial applications.

## Author contributions

Amrita Sikder: conceptualization (equal); data curation (lead); methodology (lead); writing original draft (lead). Amanda K. Pearce: data curation (supporting); editing draft (supporting); C. M. Santosh Kumar: data curation (supporting); editing draft (supporting); Rachel K. O'Reilly: conceptualization (equal); funding acquisition (supporting); supervision; writing, review & editing (supporting).

## Conflicts of interest

There are no conflicts to declare.

## Supplementary Material

MH-010-D2MH01117D-s001
