# Correction: Elucidating the role of multivalency, shape, size and functional group density on antibacterial activity of diversified supramolecular nanostructures enabled by templated assembly

**DOI:** 10.1039/d3mh90020g

**Published:** 2023-04-26

**Authors:** Amrita Sikder, Amanda K. Pearce, C. M. Santosh Kumar, Rachel K. O’Reilly

**Affiliations:** a School of Chemistry, University of Birmingham Birmingham B15 2TT UK r.oreilly@bham.ac.uk; b Institute of Microbiology and Infection, School of Biosciences, University of Birmingham Birmingham B15 2TT UK

## Abstract

Correction for ‘Elucidating the role of multivalency, shape, size and functional group density on antibacterial activity of diversified supramolecular nanostructures enabled by templated assembly’ by Amrita Sikder *et al.*, *Mater. Horiz.*, 2023, **10**, 171–178, https://doi.org/10.1039/D2MH01117D.

The authors wish to rectify an error in the published article: in Table 1, the ITC values of long cylinders and short cylinders were inadvertently interchanged. The corrected version of Table 1 is shown here.

**Table 1:** Energy of interactions of different nanoparticles with a bacterial membrane mimic as obtained by ITC

Nanoparticle	*K*_a_ (×10^−3^ M^−1^)	Δ*G* (kcal M^−1^)	Δ*H* (kcal M^−1^)	Δ*S* (cal M^−1^)
Short cylinder	2.6 ± 0.1	−9.2	−4.7 ± 0.07	15.6
Long cylinder	1.4 ± 0.03	−8.8	−4.7 ± 0.11	14.0
Nanoribbon	0.8 ± 0.1	−8.3	−4.8 ± 0.08	12.2
Sphere	0.4 ± 0.1	−6.3	−2.6 ± 0.06	9.6

The Royal Society of Chemistry apologises for these errors and any consequent inconvenience to authors and readers.

## Supplementary Material

