# Supplementary material for: Elucidating the role of multivalency, shape, size and functional group density on antibacterial activity of diversified supramolecular nanostructures enabled by templated assembly
Source: Mater Horiz. 2022 Oct 24;10(1):171–8. doi: 10.1039/d2mh01117d (PMC9810103; doi:10.1039/d2mh01117d)
Supplement: MH-010-D2MH01117D-s001 [file MH-010-D2MH01117D-s001.pdf]

Electronic Supplementary Material (ESI) for Materials Horizons.
This journal is © The Royal Society of Chemistry 2022

Supporting Information for

Elucidating the role of multivalency, shape, size and functional group density on antibacterial activity of diversified supramolecular nanostructures enabled by templated assembly

Amrita Sikder[a], Amanda K. Pearce[a], C M Santosh Kumar[b] and Rachel K. O'Reilly[a]*

[a] School of Chemistry, University of Birmingham, Edgbaston, B15 2TT, Birmingham, UK

[b] School of Bioscience, University of Birmingham, Edgbaston, B15 2TT, Birmingham, UK

*Email: r.oreilly@bham.ac.uk

Materials and Methods: All chemicals and reagents were purchased from either Sigma Aldrich, Fisher Chemicals, Acros Chemicals or Alfa Aesar and used as received. Solvents were purchased from Fisher Scientific and used as received. Dry solvents were used directly from a drying and degassing solvent tower delivery system. NMR spectra were recorded on a Bruker Avance 300, a Bruker Avance III HD 400 or a Bruker Avance III HD 500 spectrometer at 298 k and 300, 400 and 500 MHz, respectively. Shifts are quoted in δ in parts per million and quoted relative to the internal standard trimethylsilane (TMS). High Resolution Mass Spectra (ESI-MS) were conducted on a Bruker UHR-Q-ToF MaXis spectrometer with electrospray ionization. Infrared spectra were recorded in transmittance mode on an Agilent 660-IR instrument using liquid cell holder. UV-Vis spectroscopy was carried out on an Agilent Cary 60 UV-Vis Spectrometer at room temperature unless specified. Fluorescence spectra were recorded using an Agilent Cary Eclipse Fluorescence spectrophotometer. Atomic force microscopy (AFM) imaging and analysis were performed on an Asylum Research MFP3D-SA atomic force microscope in tapping mode. Samples for AFM analysis were prepared by drop casting 5 μL of solution onto a silicon wafer that had been freshly cleaned with water and ethanol, then activated using plasma treatment to generate a hydrophilic surface. Transmission electron microscopy (TEM) observations were performed on a JEOL 2000FX electron microscope at an acceleration voltage of 200 kV. All TEM samples were prepared on carbon-coated carbon grids without staining. Generally, a drop of sample (10 μL) was pipetted on a grid, blotted immediately and left to air dry. TEM images

were analyzed using the ImageJ software, and over 100 particles were counted for each sample to obtain either number-average length of the cylindrical micelle or the width of the nanoribbons.

Synthesis and Characterization: Syntheses of M1 and M2 were achieved in multiple steps using previously published procedure. PY was synthesized following Scheme S1. PY has been characterized by ^{1}H NMR spectroscopy, ^{13}C NMR spectroscopy, ESI-MS mass spectroscopy and extinction coefficient.

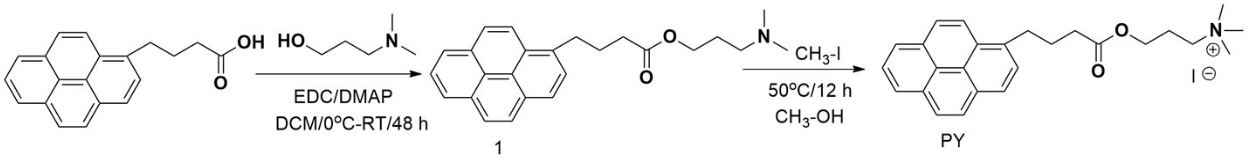

Scheme S1. Synthesis of **PY**.

Compound 1: 1-Pyrenebutyric acid (0.300 g, 1.02 mmol), 3-(dimethylamino)propan-1-ol (0.105 g, 1.02 mmol) and 4-dimethylaminopyridine (0.190 g, 1.52 mmol) were dissolved in 10 mL of dry CH_2Cl_2 taken in a round bottom flask. The reaction mixture was stirred for 30 minutes at 0 ^{o}C, then to it 1.1 equivalent of N-(3-dimethylaminopropyl)-N′-ethylcarbodiimide hydrochloride (0.242 g, 1.53 mmol) was added and it was stirred for 48 hours at room temperature. After the reaction was over, the compound was extracted with CH_2Cl_2 (15 mL) and washed with 4N HCL (15 mL), then with $NaHCO_3$ (15 mL) solution and finally with brine solution (15 mL). CH_2Cl_2 solution was passed through anhydrous Na_2SO_4 and evaporated the solvent to get crude product. It was purified by column chromatography using CH_2Cl_2/ CH_3OH (95:5) solvent mixture as an eluent to get the pure compound as white solid (product obtained-0.260 g, yield-68%). ^{1}H NMR (300 MHz, $CDCl_3$): δ (ppm) = 8.35-7.88 (9H, m), 4.16 (2H, t, J = 6 Hz), 3.42 (2H, t, J= 6 Hz), 2.48 (2H, t, J = 6 Hz), 2.25 (2H, t, J = 6 Hz), 2.21 (6H, s), 2.20-2.17 (2H, m), 1.86-1.83 (2H, m).

PY: Compound **1**(0.200 g, 0.074 mmol) and large excess of methyl iodide (0.5 mL) were taken in a sealed tune dissolved in 5 mL of CH_3OH. The reaction mixture was stirred at 50°C for 24 h. After reaction excess methyl iodide and CH_3OH were evaporated under reduced pressure to obtain yellow solid. This was purified by column chromatography using CH_2Cl_2/ CH_3OH (90:10) solvent mixture as an eluent to get the pure compound as white solid (product obtained-0.178 g, yield-62%). ^{1}H NMR (300 MHz, CD_3OD): δ (ppm) = 8.42-7.92 (9H, m), 4.14 (2H, t),

3.47-3.42 (4H, m), 3.0 (9H, s), 2.53 (2H, t, J=6H), 2.26-2.17 (2H, m), 2.12- 2.03 (2H, m). ^{13}C NMR (DMSO-d6): 173.23, 136.56, 131.37, 130.89, 129.89, 128.67, 128.07, 127.94, 127.81, 127.10, 126.70, 125.52, 125.46, 125.34, 124.73, 124.61, 123.91. HRMS (ESI): m/z calculated for $C_{26}H_{30}NO_2$: 388.228; found: 388.230. UV-Visible (THF): λ_{max}= 342 nm (23100 $M^{-1}cm^{-1}$), 326 nm (15600 $M^{-1}cm^{-1}$), 311 nm (6500 $M^{-1}cm^{-1}$).

Experimental Procedures:

Sample preparation: Amphiphile M1 and M2 are molecularly dissolved in THF and does not form any assembly in this solvent. Whereas cationic amphiphile PY is molecularly dissolved in CH_3OH. Therefore, stock solutions of M1/M2 were made in THF (2.0 mM) and stock solution of PY was made in CH_3OH (2.0 mM). To prepare the nanomaterials in aqueous solution, a measured volume of each stock (both M1/M2 and PY) solution was taken in a vial and the solvents were completely evaporated by heating (≤ 70°C) to obtain a dry thin film. Measured amount of miliQ water was added to the vial to dissolve the film. To monitor charge transfer band a 5 mM solution was prepared. This was then diluted to 0.5 mM for doing other experiments such as fluorescence spectroscopy, TEM. The 0.5 mM solution was further half-diluted to 0.25 mM for obtaining clearer image in AFM. The solutions were allowed to equilibrate for 1 hour at room temperature before any physical studies and considered as freshly prepared solutions. Further, the M2+PY solutions both 5 mM concentration and 0.5 mM concentration were aged for 5 weeks at ~20 °C to perform experiments with aged solutions unless specified. For dry state TEM sample preparation: 0.5 mM aqueous solution was prepared without any staining agent**.** For AFM 0.5 mM solution was diluted to 0.25 mM prior to deposition on the mica surface. This is to be noted that, different concentration has been used for TEM and AFM to obtain clearer images of the nanoparticles without much overlapping, and the difference in concentration does not alter the morphology of the nanoparticles.

Further truncated cylinders were prepared by sonicating the aqueous solution of M1+PY (0. 5 mM) using a sonicate bath at 20oC. The sonication time was changed between 15-50 minutes (done at 15 minutes, 25 minutes, 35 minutes and 50 minutes to obtain cylinders of length 2.5-3.5 μm, 1.6-2.5 μm, 1.6-1.0 μm and 0.6-0.9 μm respectively) keeping all the other parameter same (temperature, concentration of the solution).

Molar extinction coefficients of PY was determined from the Lambert Beer equation. For co-assembly study, solvent dependent UV-Vis spectra were taken using 0.1 cm quartz cuvettes. 0.5 mM solution was used for recording UV-Vis and fluorescence spectra 0.5 mM.

Determination of association constant: UV-Vis spectra of 1:1 (M1+PY or M2+PY) solution were recorded as a function of concentration at a fixed temperature. Ka was determined by using equation 1, where c, A, l and Ɛ defines concentration, absorbance, optical path length and extinction coefficient, respectively.

$$\frac{C}{A} = \frac{1}{\sqrt{K\varepsilon l}} \times \frac{1}{\sqrt{A}} + \frac{1}{\varepsilon l} \text{ .... (1)}$$

Tuning cylinder length: 5 set of 3:2 M1:PY aqueous solution (2 mM) were prepared from the same stock solutions. Each vial was sonicated for different time (0 min, 15 min, 25 min, 35 min, 50 min). Each solution was drop casted on TEM grid to check the cylindrical length. TEM images were analysed by ImageJ software. The length of the cylinders was obtained by counting at least 100 particles for each sample. Freshly sonicated cylinders were used for MIC evaluation.

MIC determination: Bacteriostatic activities were evaluated using a broth microdilution minimum inhibitory concentration (MIC) assay. Gram-positive *Staphylococcus aureus* bacteria and Gram-negative *Escherichia coli* bacteria were cultivated overnight at 37 °C. Stock solutions of nanoparticles were diluted two-fold with LB broth across seven wells of a 96-well plate to give a range of concentrations from 1000 to 16 μM and a volume of 100 μL. Then, 100 μL of bacterial suspension (*S. aureus*, 5 × 10^7 CFU mL-1; *E. coli*, 3 × 10^7 CFU mL^{-1}) was placed in each well. In each row, one well contained bacteria but no compound, and one well contained LB broth but no bacteria to act as controls. The 96-well plates were cultured on a shaking bed at 150 rpm and 37 °C for 24 h, followed by the measurement of the optical density (OD) at a wavelength of 700 nm, where the MIC was taken at the concentration where no growth was observed with a visible spectrophotometer in the growing phase of the microorganisms. The wells with broth containing bacteria alone were used as the comparison growth rate, and the tests were repeated three times. Although the OD value is not linearly related to the bacterial quantity, it is well reported to provide evidence of antibacterial activity. [1]

Zone inhibition assay: Zone inhabitation assay has been done following well established procedure.[2] The antimicrobial nanoparticles were diluted serially in sterile distilled water to

obtain 0.25, 0.5, 1, and 2 mM (used 1:1 ratio, concentration calculated with respect to PY). *S. aureus* (ATCC 29213) was grown overnight in Luria Bertoni broth at 37 °C. The bacterial culture was spread on the LB agar plates using cotton swabs. Sterile filter-disks were placed on the agar plates. 10 µl of the serially diluted nanoparticle solutions were spotted onto the filter discs and the plates were incubated at 37 °C overnight.

Bacterial viability assay: Viability of *S. aureus* upon treatment with the cylindrical nanoparticles was assessed using a fluorescence-based double staining bacterial viability assay. [3] To the log phase cultures of *S. aureus,* the cylindrical nanoparticles were added at the sub-MIC concentrations (10 and 20 µM) and the cultures were incubated at 37 °C for 4 h. The bacterial cells were recovered by centrifugation and were washed three times with 1x PBS to remove the residual nanoparticles. The cells were labelled using the LIVE/DEAD® BacLight™ Bacterial Viability Kit for Microscopy (Molecular Probes, Inc., Eugene, OR, USA), following the manufacturer's instructions. Briefly, equal volumes of the kit components A and B, containing DNA intercalating dyes SYTO 9 and Propidium iodide, were mixed in a microfuge tube. 3 µL of the mixture was added to one mL bacterial suspension and incubated at the room temperature in the dark for 15 minutes. 5 µL of the stained bacterial suspension was trapped between the slides and 18 mm square coverslips. The labelled cells were examined using the Zeiss Axio Observer inverted fluorescent microscope with a 100-X objective. Untreated bacterial cultures were used as controls.

TEM image of the bacteria: The effect of the cylindrical nanoparticles on *S. aureus* membranes was assessed using transmission electron microscopy, following the reported protocols.[4] To the log phase cultures of *S. aureus,* the cylindrical nanoparticles were added to a final concentration of 10 µM and the cultures were incubated at 37 °C for 12 h. The bacterial cells were recovered by centrifugation and were washed three times with 1 × PBS to remove the residual nanoparticles. The cells were fixed with 4% w/v paraformaldehyde solution. The fixed cells were washed with 1 × PBS to remove the residual fixing solution. The cells were suspended in 1 × PBS and 5 µL of bacterial suspension was placed on the copper grids. Cells were dried and images were recorded in a high-resolution on a JEOL 2000FX electron microscope at an acceleration voltage of 200 kV.

Haemolysis assay: The toxicity of the antimicrobial agents was assessed using their ability to lyse the red blood cells in blood agar plate assay[4]. Blood agar plates were prepared by mixing 4 ml of defibrinated horse blood (E & O Laboratories Ltd., Bonnybridge, UK) and 50 ml 4% w/v blood agar base (Oxoid Ltd., Basingstoke, UK). The 4 mM stock solutions of antimicrobial nanoparticles were spotted onto the plates and the plates were incubated at 37 °C overnight. 10 mM SDS was used as the haemolysing positive control.

ITC experiment: Interaction of the nanoparticles with lipids were performed at 25°C in aqueous solution. 1.0 mM of aqueous solution of lipid vesicles (POPC or POPG/POPE (3:1)) were prepared using standardised protocol[5]. Aqueous solution of nanoparticles (0.1 mM with respect to PY concentration) with different shape and size was prepared using 1:1 ratio of M1/M2 and PY. The lipid solution was taken in the syringe, and the nanoparticle solution (0.1 mM) was filled in the calorimetric cell. The experiment consisted of 19 injections of 2.0 µL each with 120 sec intervals with a stirring speed of 500 rpm to ensure that the titration peak returned to the baseline before the next injection was carried out. A background titration was performed under the same conditions with water placed in the calorimetric cell instead of the nanoparticle solution. Background data was subtracted from each sample titration to eliminate the heat of dilution. The titration curves were analyzed using the "one-binding-site" model to determine the binding constant (Ka), enthalpy of binding (ΔH). The Gibbs free energy of binding (ΔG) and entropy of binding (ΔS) were calculated using the equations.

$$\Delta G^\circ = -RT\ln K_{ap} \quad \text{……….(2)}$$

$$\Delta S^\circ = (\Delta H^\circ - \Delta G^\circ)/T \quad \text{……...(3)}$$

Table-1: MIC (μM concentration) value screened for different nanoparticles

% of PY	MIC with respect to total nanoparticle (μM)					
	Cylindrical micelle		Spherical Micelle		Nanoribbon	
	S. aureus	*E. coli*	*S. aureus*	*E. coli*	*S. aureus*	*E. coli*
0	-	-	-	-	-	-
10	-	-	-	-	-	-
20	500	1000	-	-	-	-
30	250	500	500	1000	500	-
40	125	500	500	1000	250	1000
50	125	500	500	1000	250	1000

Table-2: MIC (μM concentration) with respect to PY present for different nanoparticles

% of PY	MIC with respect to PY (mM)					
	Cylindrical micelle		Spherical Micelle		Nanoribbon	
	S. aureus	*E. coli*	*S. aureus*	*E. coli*	*S. aureus*	*E. coli*
0	-	-	-	-	-	-
10	-	-	-	-	-	-
20	100	200	-	-	-	-
30	75	150	150	300	75	-
40	50	200	200	400	100	400
50	62.5	250	250	500	125	500

Table-3: MIC (μg/mL concentration) with respect to PY present for different nanoparticles

% of PY	MIC with respect to PY(μg/mL)					
	Cylindrical micelle		Spherical Micelle		Nanoribbon	
	S. aureus	*E. coli*	*S. aureus*	*E. coli*	*S. aureus*	*E. coli*
0	-	-	-	-	-	-
10	-	-	-	-	-	-
20	39	76	-	-	-	-
30	29	58	58	116	29	-
40	20	78	77	155	39	155
50	24	97	97	194	83	194

Additional Figures:

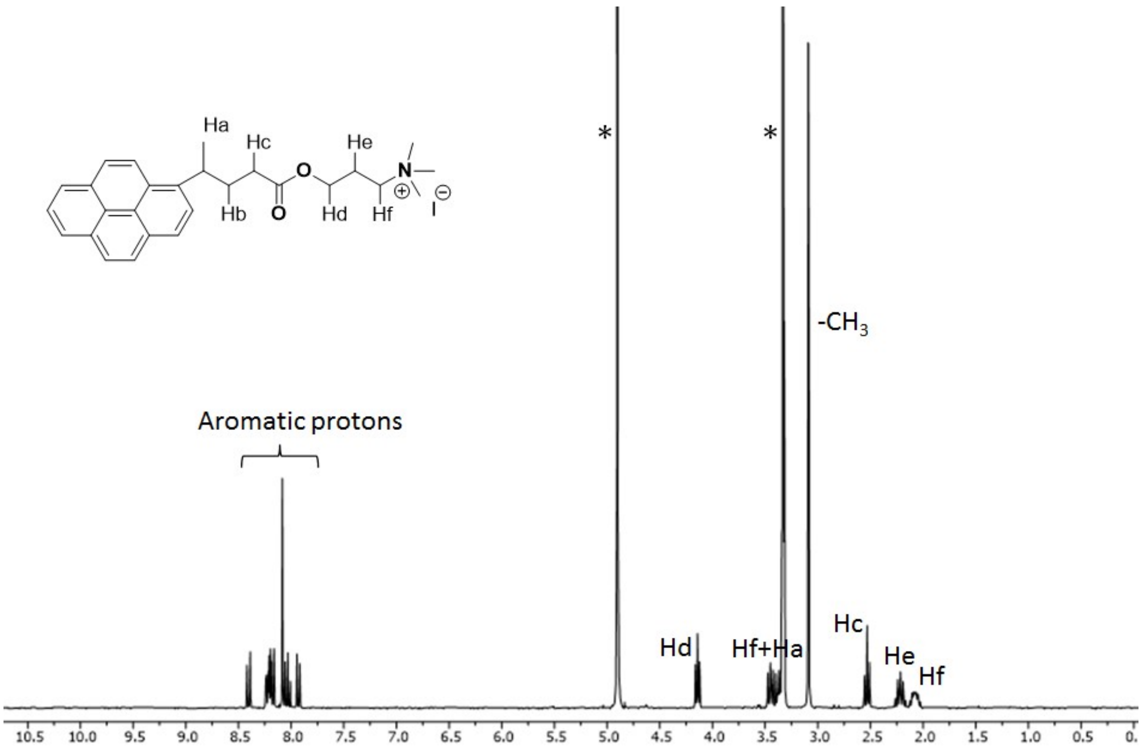

Figure S1. ^1H NMR spectroscopy of PY. Solvent Methanol-d4. * Denotes solvent peak.

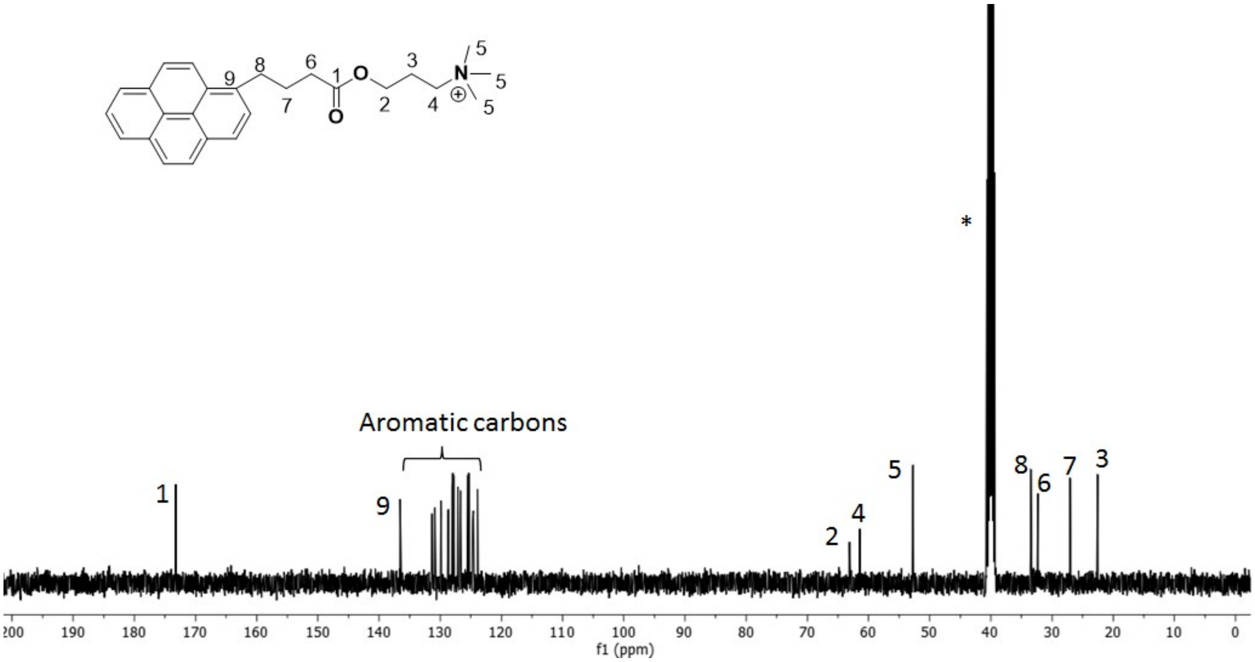

Figure S2. ^{13}C NMR spectroscopy of PY. Solvent DMSO-d6. * Denotes solvent peak.

Figure S3. a) Solvent dependent UV-Vis spectra and b) fluorescence spectra of PY;

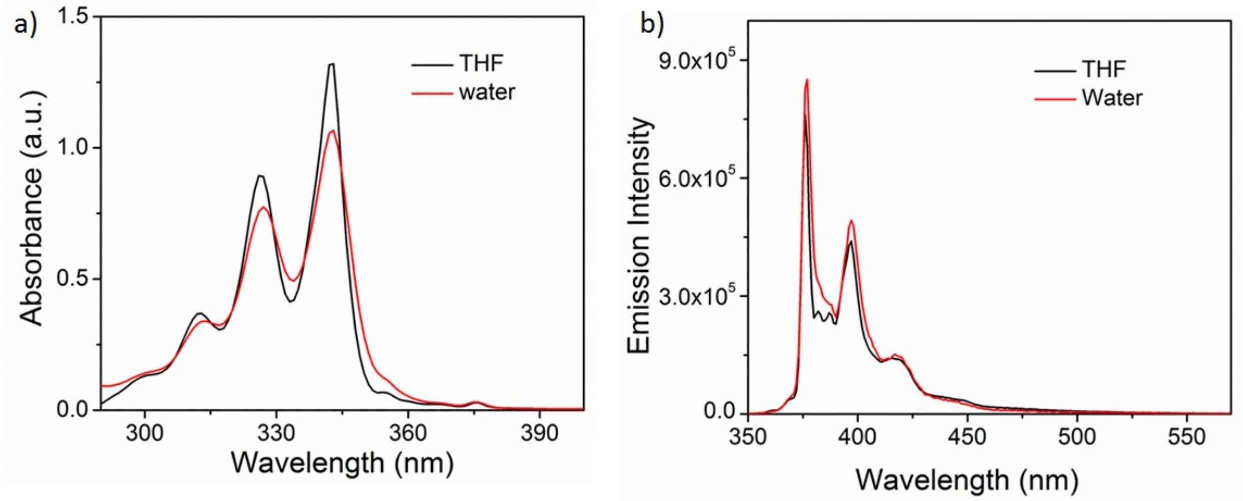

Concentration=0.5 mM, l=0.1cm, λ_{ex}=335 nm, slit-1.

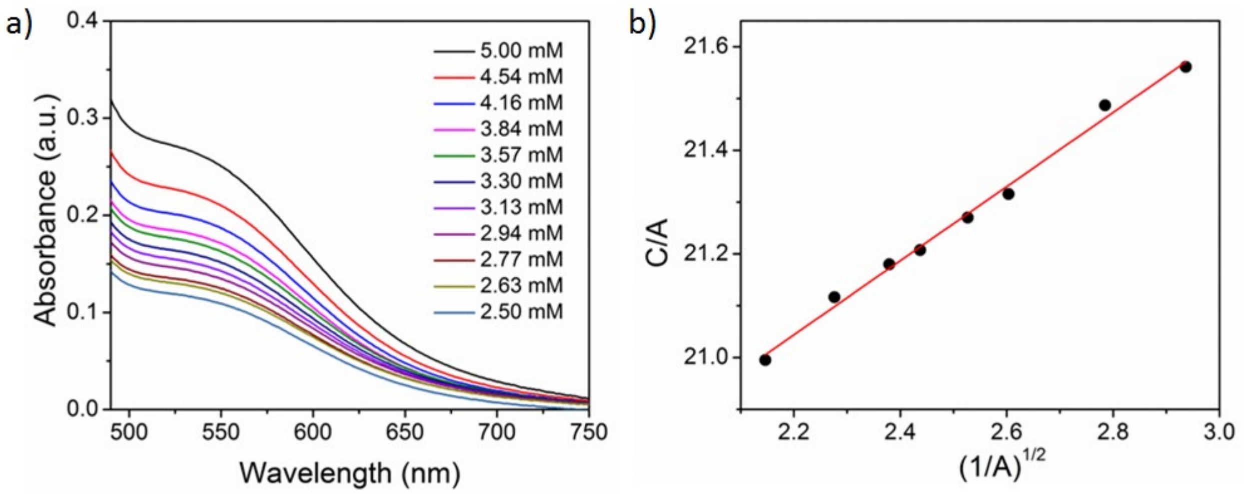

Figure S4. a) Dilution experiment for 1:1 M1+ PY; b) C/A Vs $(1/A)^{0.5}$ plot for Ka value determination. *l*=0.1 cm, T=20°C.

Figure S5. (a) UV-Vis and (b) Fluorescence spectra of aqueous solution of 1:1 mixture of M2

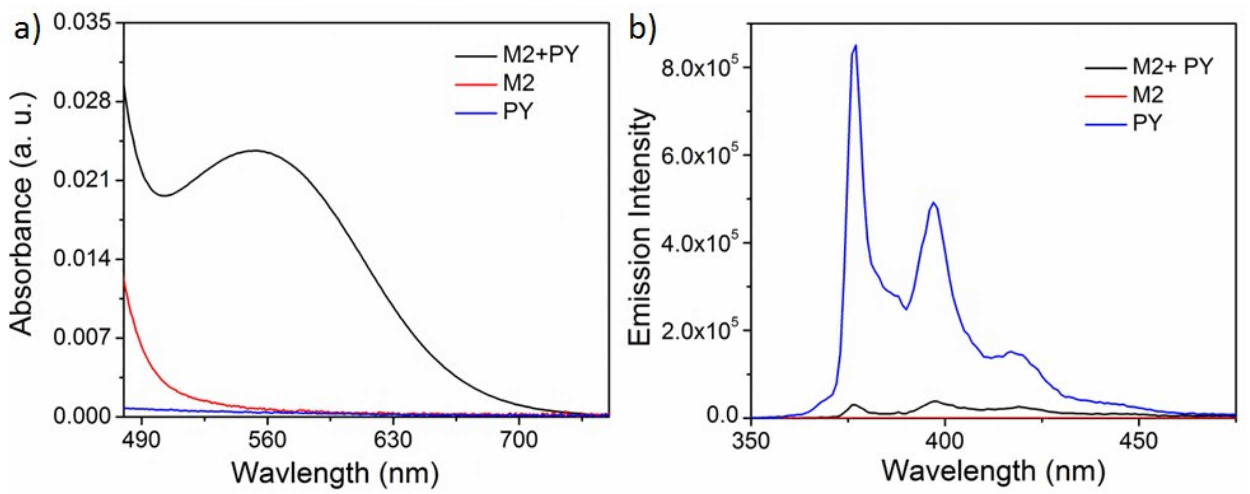

and PY (freshly prepared solution). Concentration for UV-Vis measurement=0.5 mM, *l*=0.1 cm; concentration for fluorescence measurement=0.5 mM, slit=1.

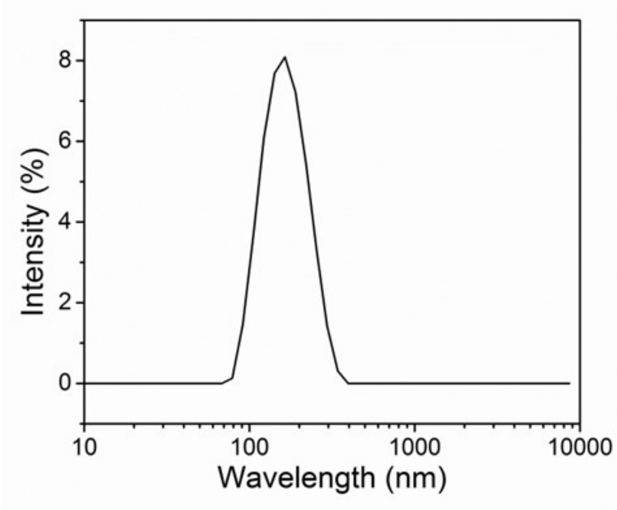

Figure S6. Particle size distribution of the micellar nanoparticle (1:1 M2+PY freshly prepared solution) obtained from DLS; concentration=0.5mM.

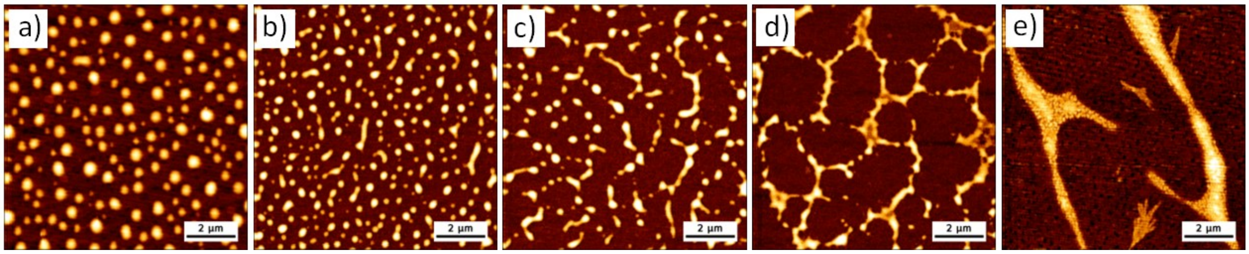

Figure S7. Time dependent AFM. Concentration=0.25 mM. (a) day-1; b) day-7; c) day-14; d)

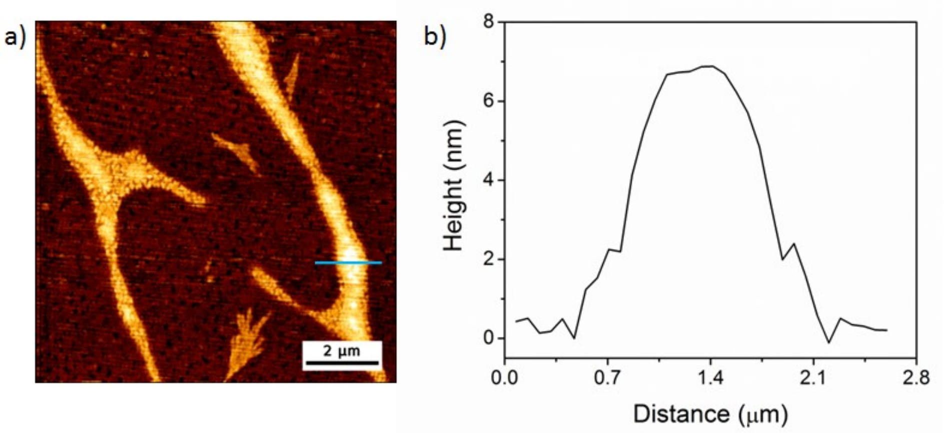

day-21; e) day-30.

Figure S8. a) AFM image of 2D-nanoribbon and b) corresponding height bar image.

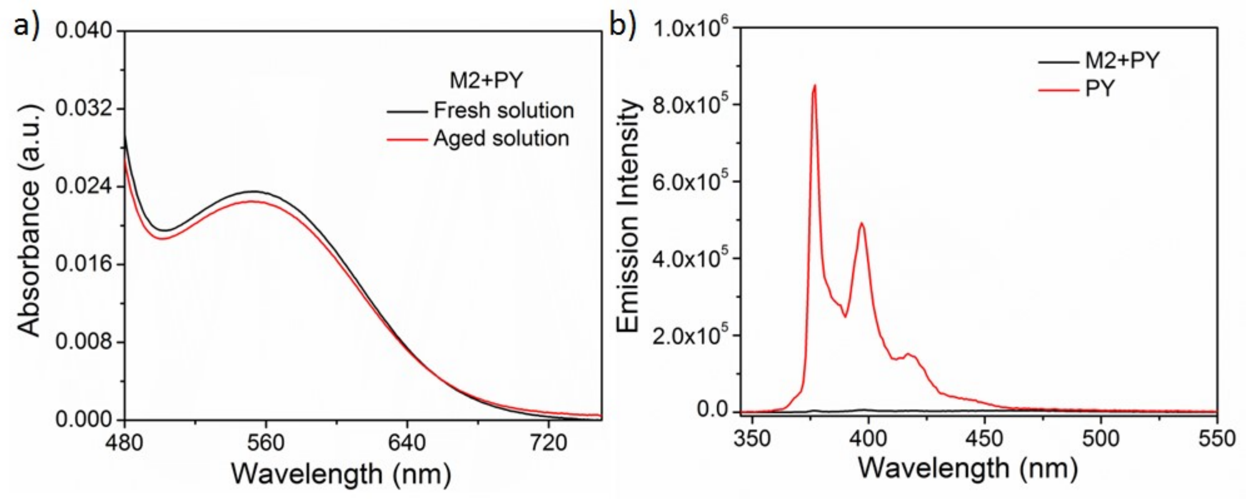

Figure S9. a) UV-Vis and (b) Fluorescence spectra of aqueous solution of 1:1 mixture of M2 and PY (aged solution). Concentration for UV-Vis measurement= 0.5 mM, *l*=0.1 cm; concentration for fluorescence measurement=0.5 mM, slit=1.

Figure S10. a) Dilution experiment for 1:1 M2+ PY (freshly prepared solution); b) C/A Vs

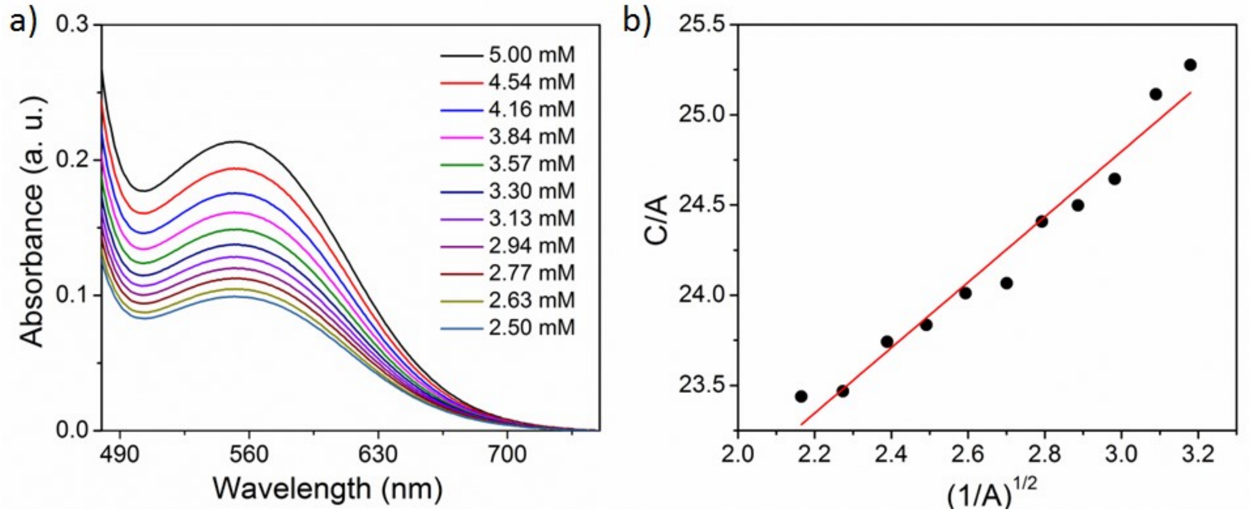

$(1/A)^{0.5}$ plot for Ka value determination. *l*=0.1 cm, T=20°C.

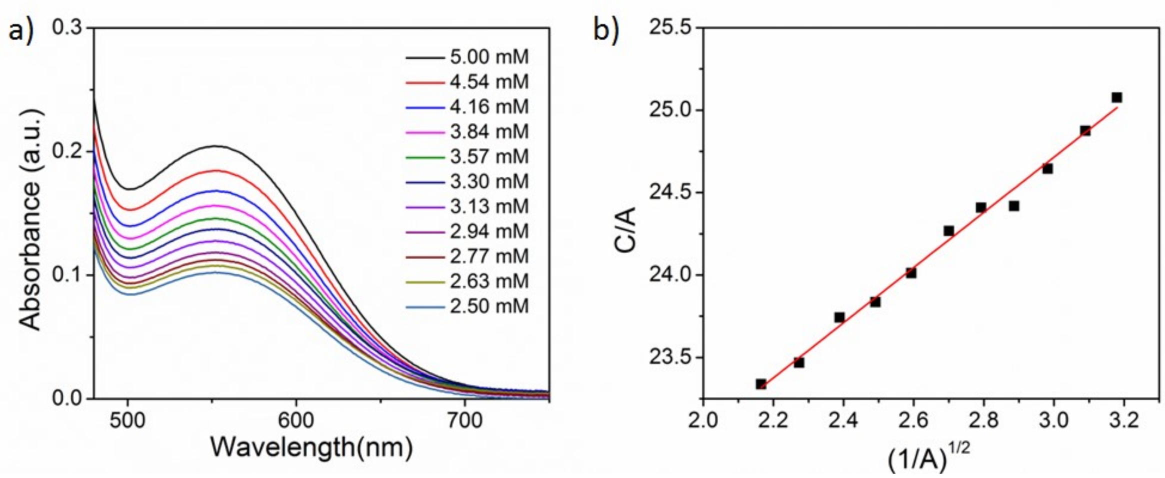

Figure S11. a) Dilution experiment for 1:1 M2+PY (aged solution); b) C/A Vs $(1/A)^{0.5}$ plot for Ka value determination. l=0.1 cm, T=20°C.

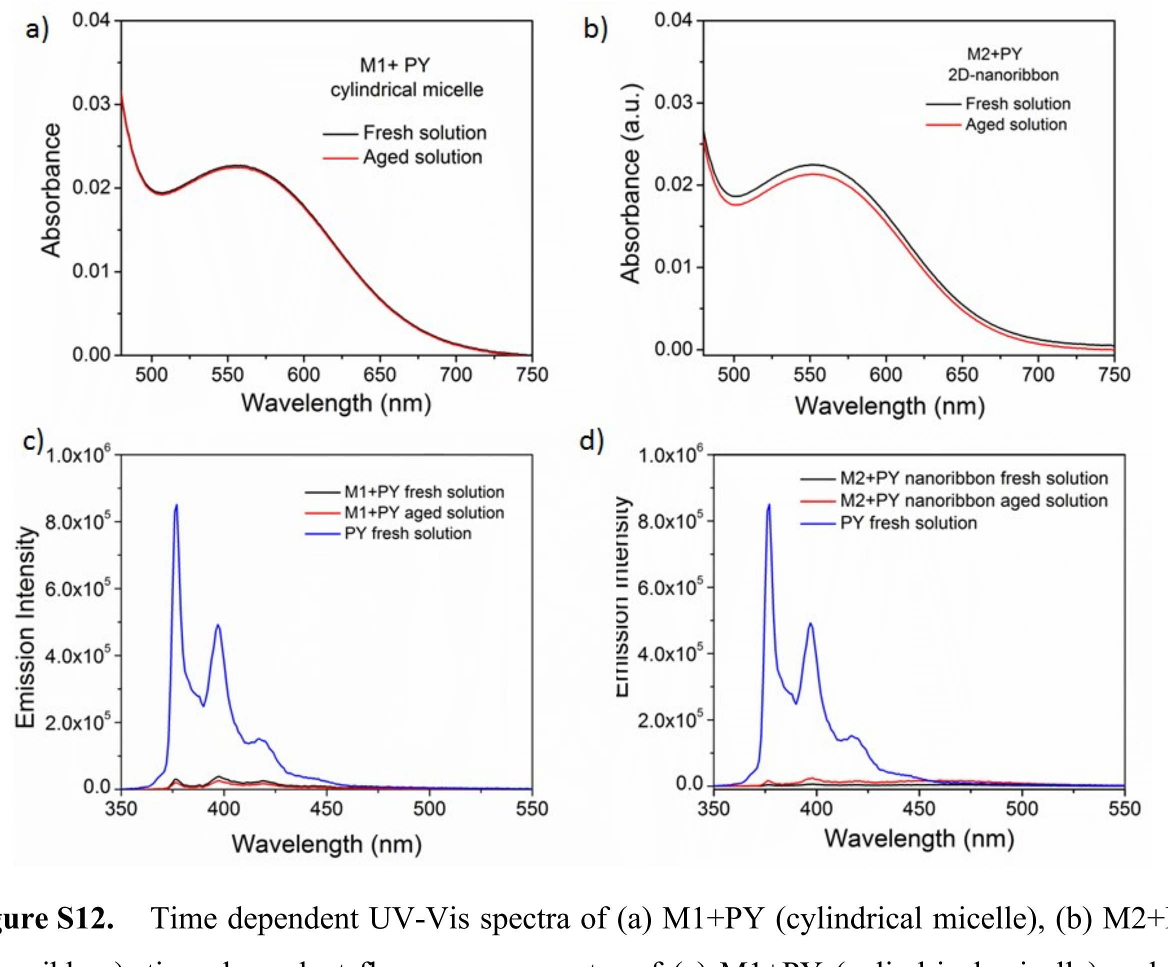

Figure S12. Time dependent UV-Vis spectra of (a) M1+PY (cylindrical micelle), (b) M2+PY (nanoribbon); time dependent fluorescence spectra of (c) M1+PY (cylindrical micelle) and (d)

M2+PY (nanoribbon). Fresh solution indicates the day nanoparticles were first prepared whereas aged solution was prepared by keeping nanoparticle solution at 20°C for 7 days.

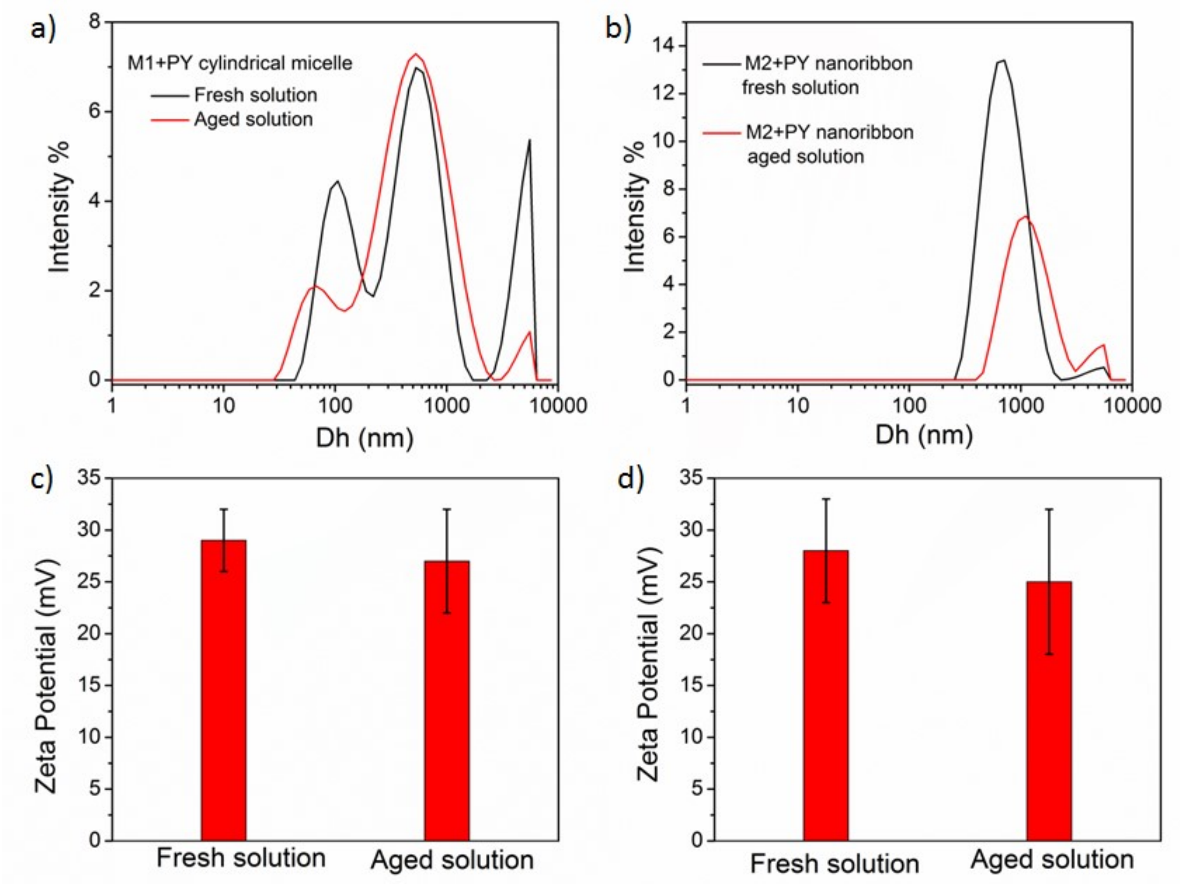

Figure S13. Time dependent DLS of (a) M1+PY (cylindrical micelle), (b) M2+PY (nanoribbon); time dependent zeta potential measurement for (c) M2+PY (cylindrical micelle), (d) M2+PY (nanoribbon). Fresh solution indicates the day nanoparticles were first prepared whereas aged solution was prepared by keeping nanoparticle solution at 20°C for 7 days.

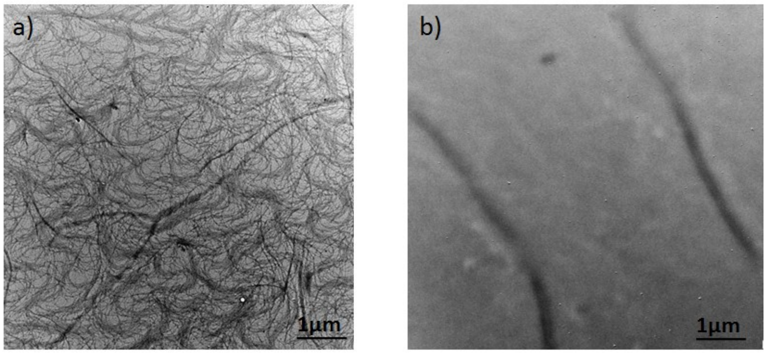

Figure S14. (a)TEM image of M1+PY (cylindrical micelle) and (b) M2+PY (nanoribbon) after 7 days.

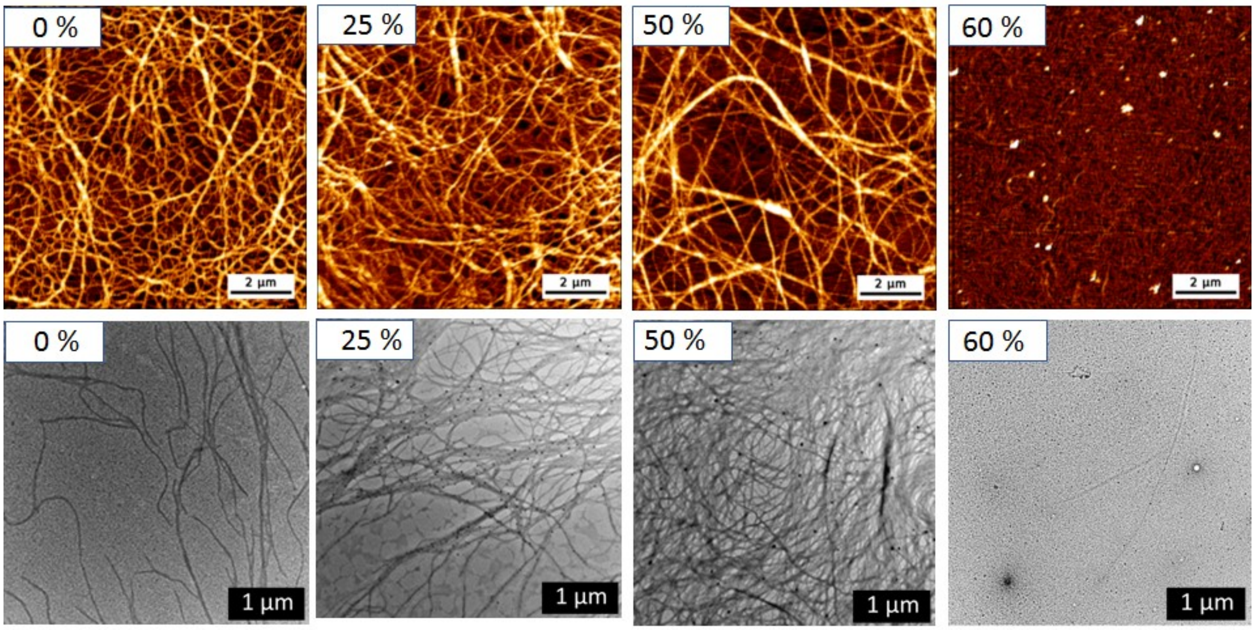

Figure S15. TEM and AFM images of M1+PY at different ratio. Concentration for TEM=0.5 mM; concentration for AFM=0.25 mM.

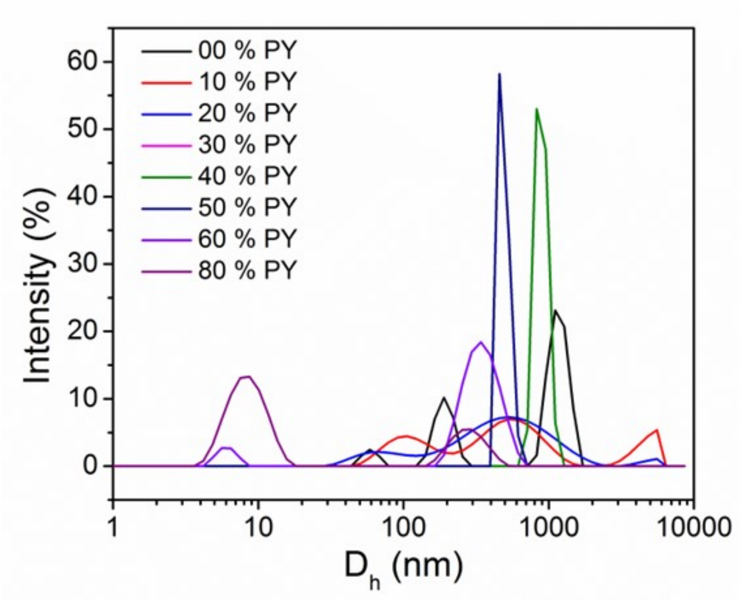

Figure S16. Particle size distribution M1+PY at different M1:PY ratio obtained from DLS; total chromophoric concentration=0.5mM. Appearance of smaller peak at higher PY % indicates disassembly.

Figure S17. Plot of zeta potential vs % of PY in each nanoparticle; concentration=0.5mM.

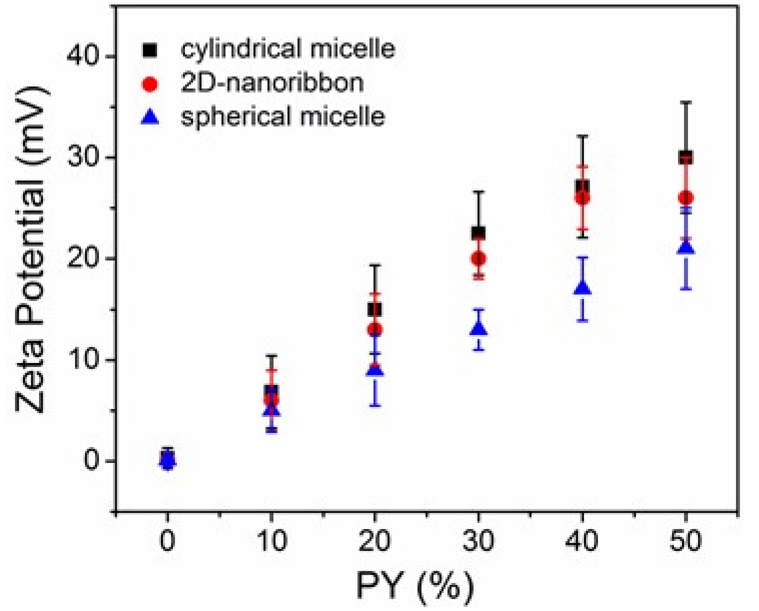

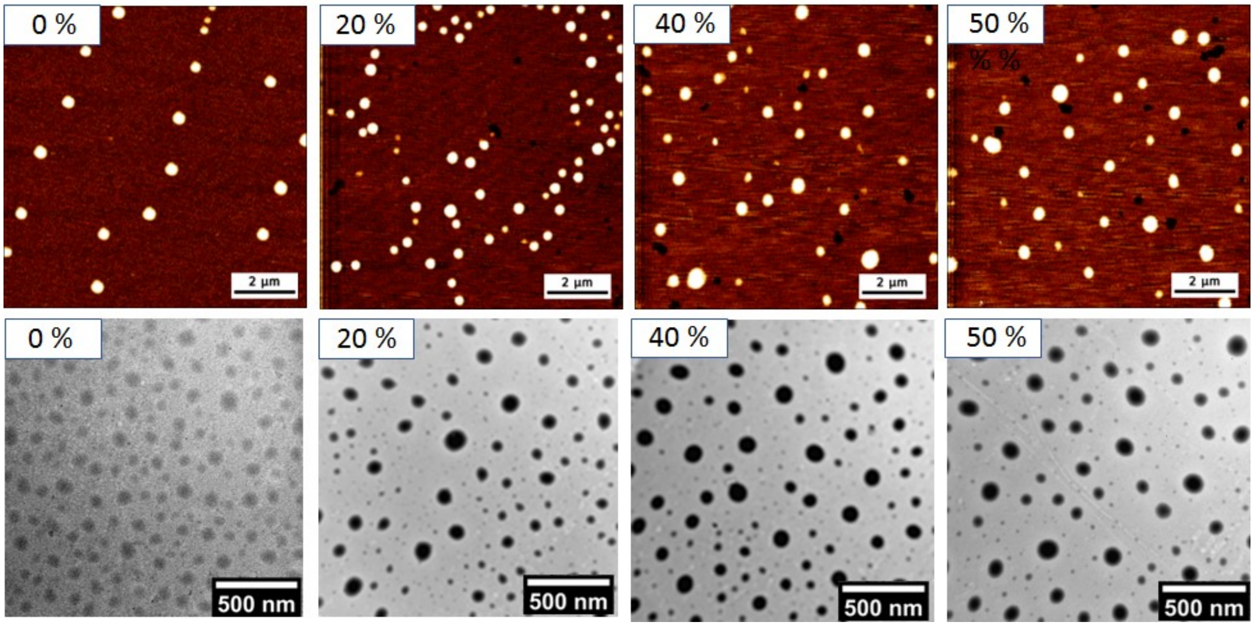

Figure S18. TEM and AFM images of M2+PY (freshly prepared solution) at different ratio. Concentration for TEM=0.5 mM; concentration for AFM=0.25 mM.

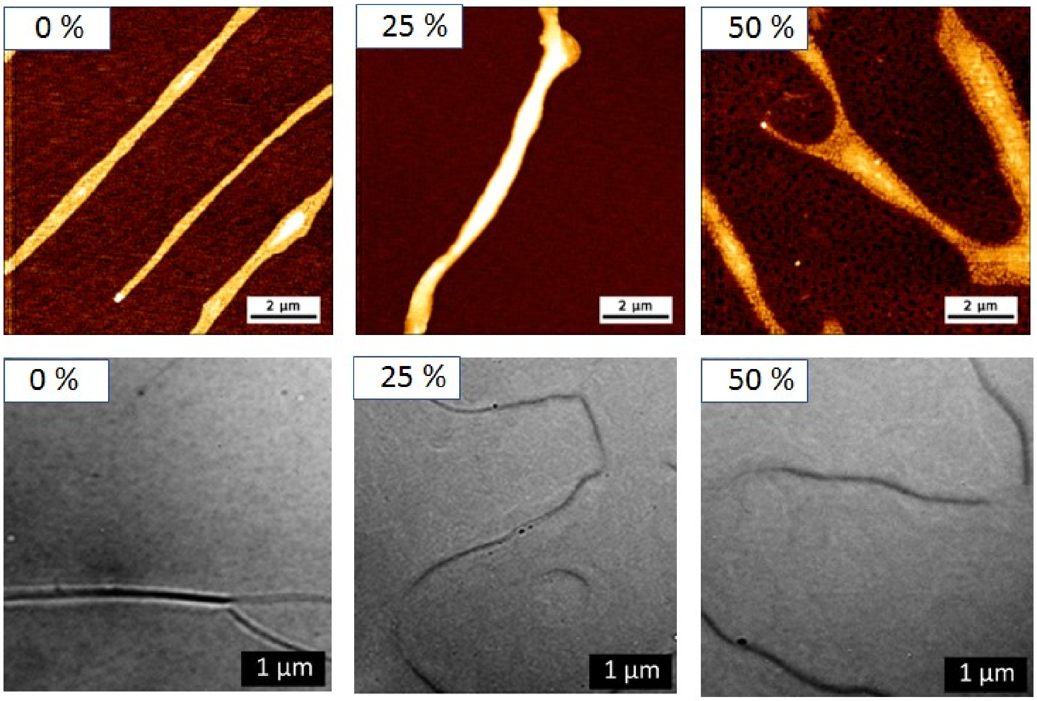

Figure S19. TEM and AFM images of M2+PY (aged solution) at different ratio. Concentration for TEM=0.5 mM; concentration for AFM=0.25 mM.

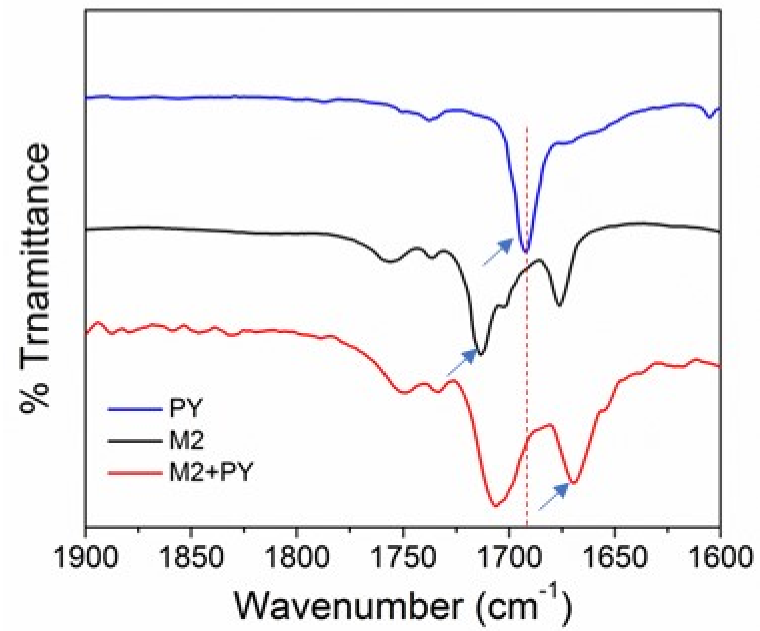

Figure S20. IR spectra of M2, PY and M2+PY in D_2O; concentration=1 mM. The arrow corresponds to >C=O stretching frequency of the ester bond for PY and amide bond for M2. Peak shift of the C=O of PY in presence of M2 indicated intermolecular H-bonding between M2 and PY.

Figure S21. a) Molecular complex consisting of M1 and PY. The red box indicates complete

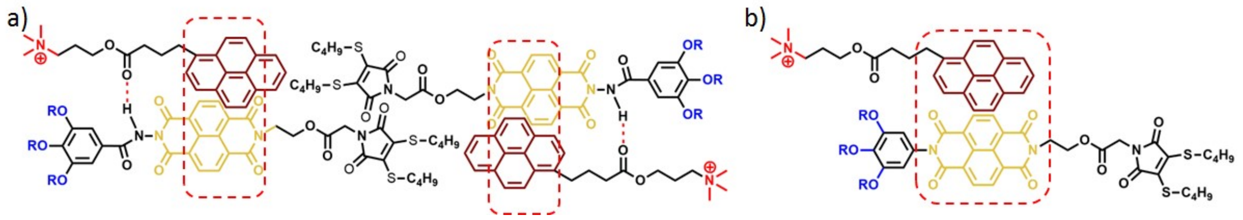

chromophoric overlap; b) Molecular complex consisting of M2 and PY. The red box indicates partial chromophoric overlap between M2 and PY.

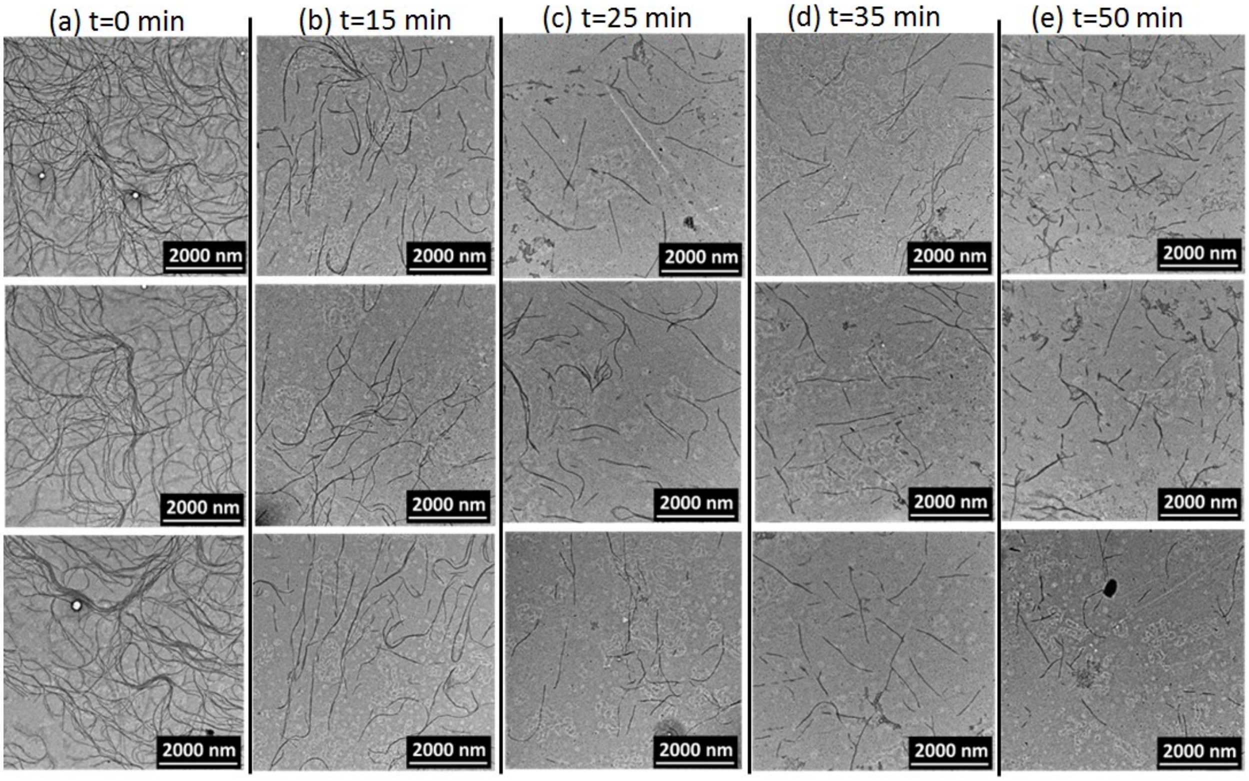

Figure S22. TEM images of cylindrical micelles of variable length prepared by sonication. Concentration=0.5 mM.

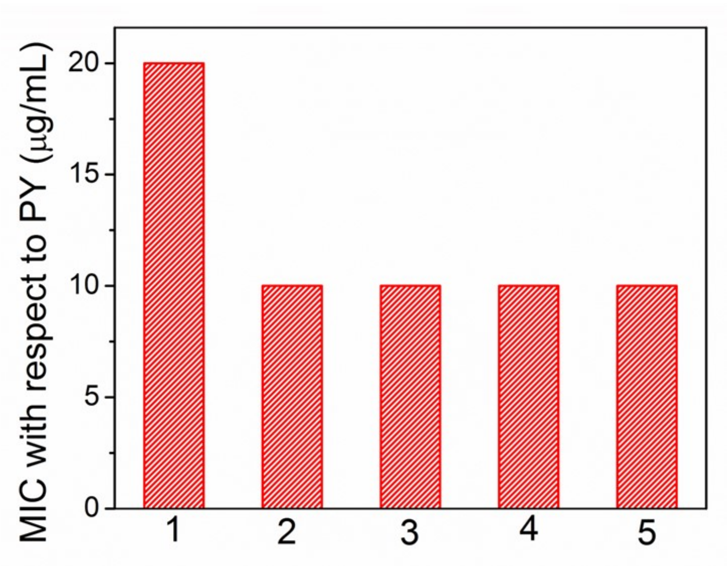

Figure S23. Bar plot of MIC vs length of the cylindrical micelles; cylinder length: 1. > 10 μm; 2. 2.5-3.5 μm; 3. 1.6-2.5 μm; 4. 1.6-1.0 μm, 5. 0.6-0.9 μm.

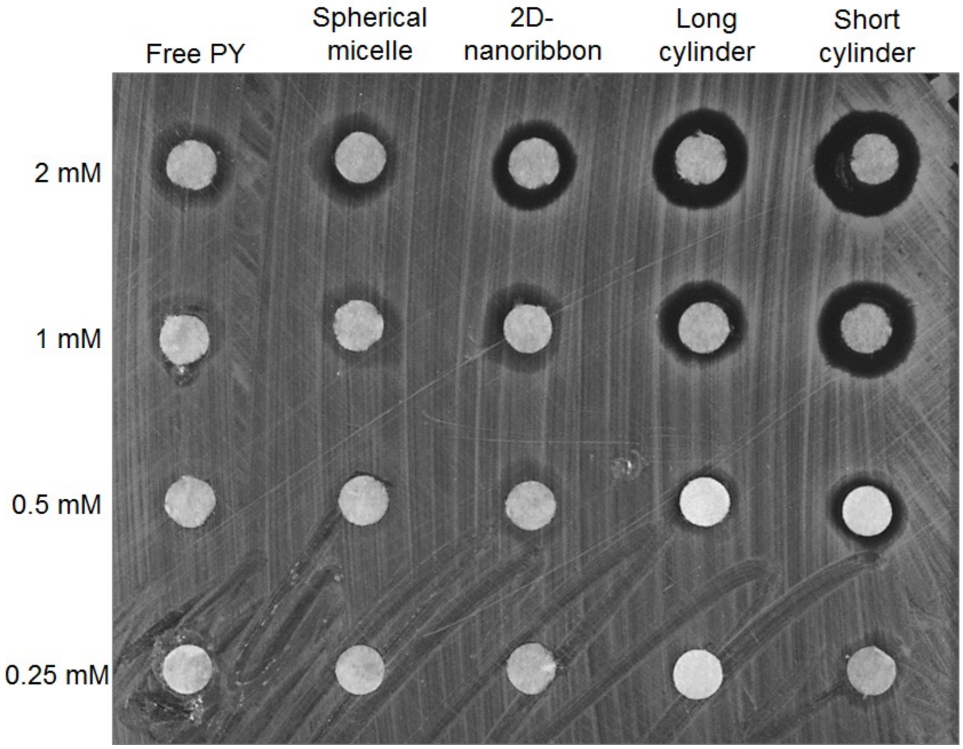

Figure S24. Representative image showing the growth inhibition by the indicated antimicrobial agents. Overnight cultures of *S. aureus* (ATCC 29213) was spread on LB agar plates. The indicated serial dilutions of the antimicrobial agents were spotted on to the sterile filter disks. The plates were imaged post overnight incubation at 37 °C.

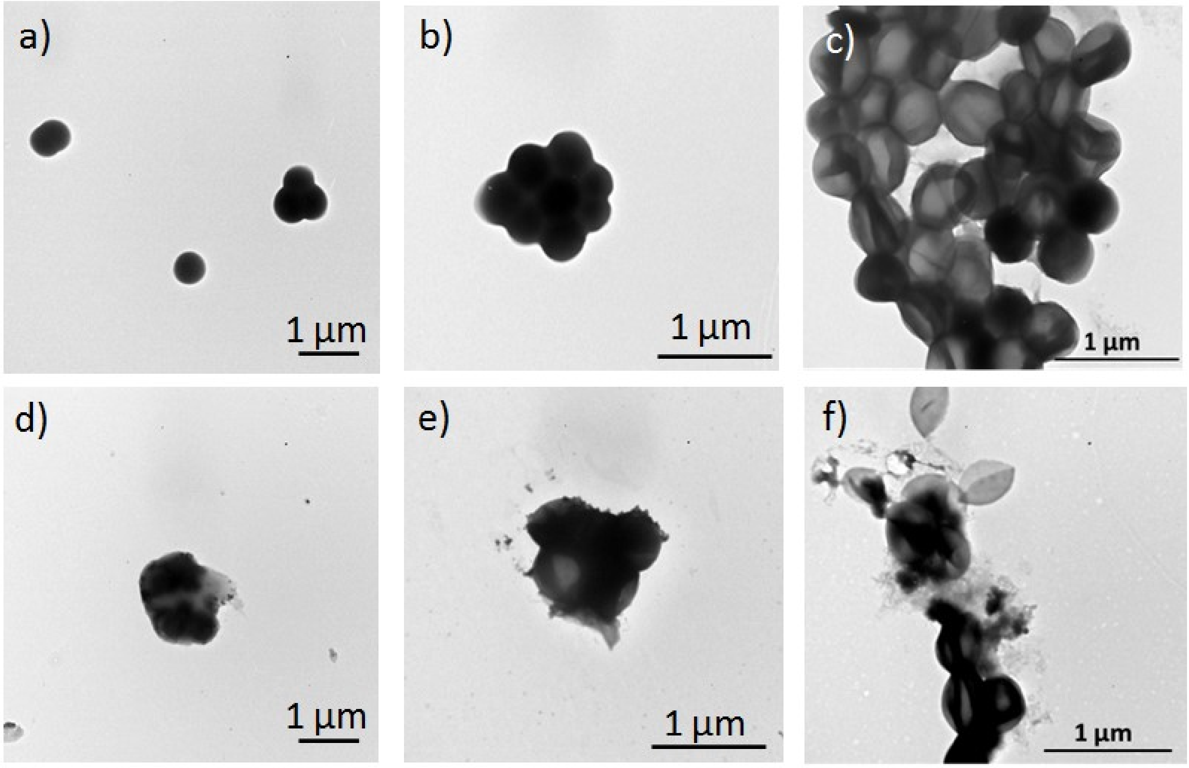

Figure S25. TEM images of 3:2 M1+PY treated (d-e) and untreated (a-c) *S. aureus* cells.

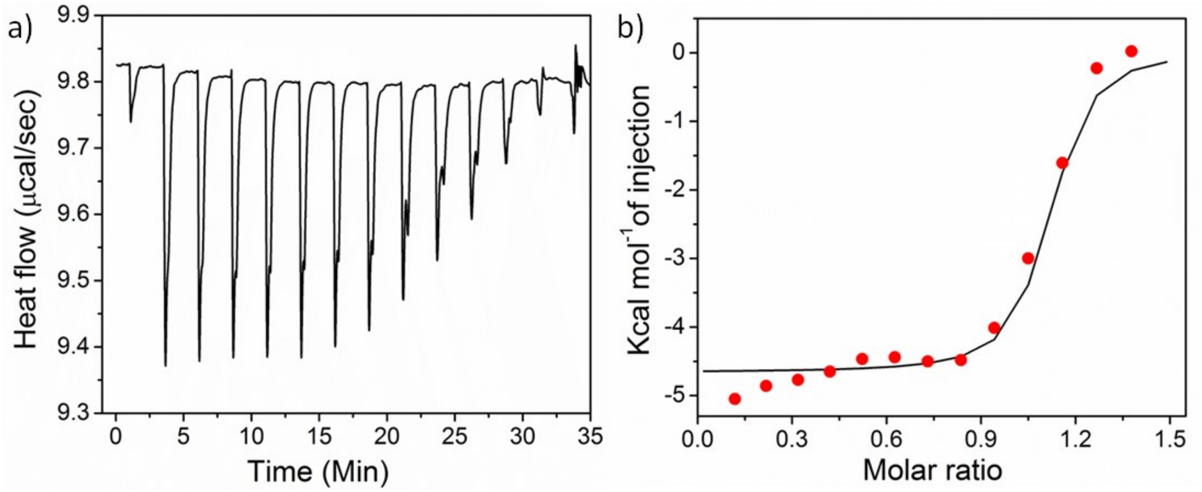

Figure S26. Isothermal titration calorimetry (ITC) thermograms of short cylindrical nanoparticles with POPG: POPE (3: 1) liposomes; b) corresponding enthalpogram. Lipid concentration is 1.0 mM and concentration of nanoparticle 0.1 mM.

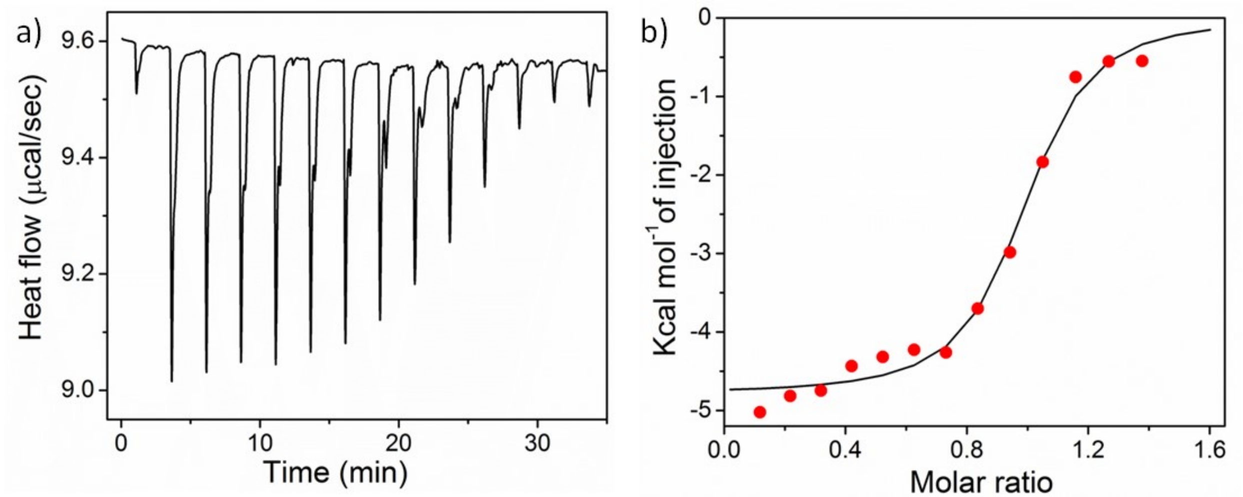

Figure S27. Isothermal titration calorimetry (ITC) thermograms of nanoribbons with POPG: POPE (3: 1) liposome; b) corresponding enthalpogram. Lipid concentration is 1.0 mM and concentration of nanoparticle 0.1 mM.

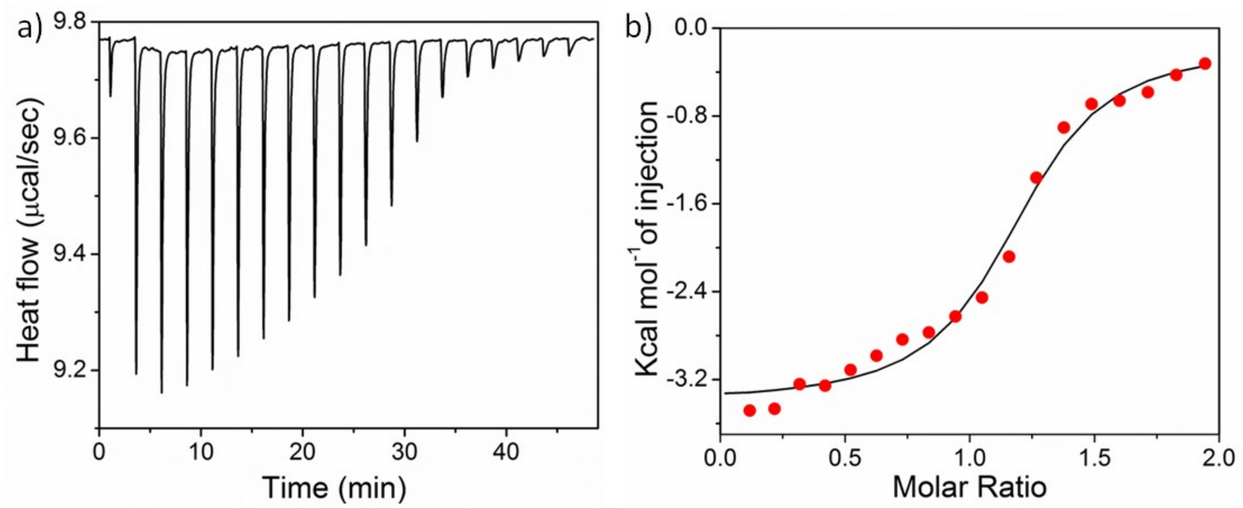

Figure S28. Isothermal titration calorimetry (ITC) thermograms of spherical micelles with POPG: POPE (3: 1) liposome; b) corresponding enthalpogram. Lipid concentration is 1.0 mM and concentration of nanoparticle 0.1 mM.

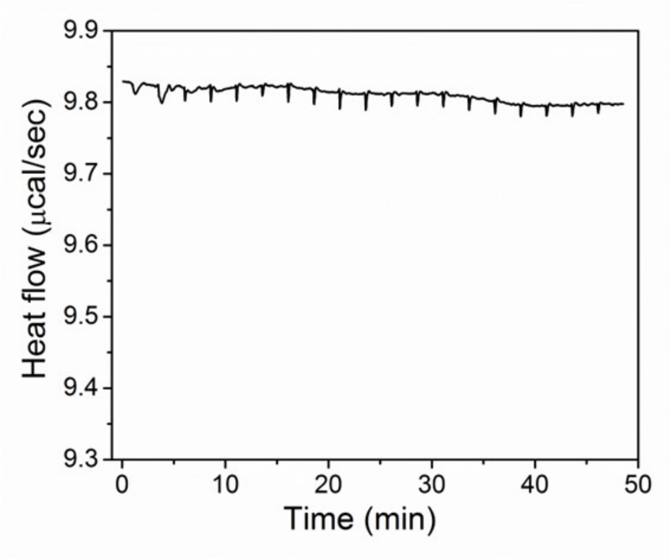

Figure S29. Isothermal titration calorimetry (ITC) thermograms of short cylindrical nanoparticles with POPC liposome (mammalian cell membrane mimic); Lipid concentration is 1.0 mM and concentration of nanoparticle 0.1 mM.

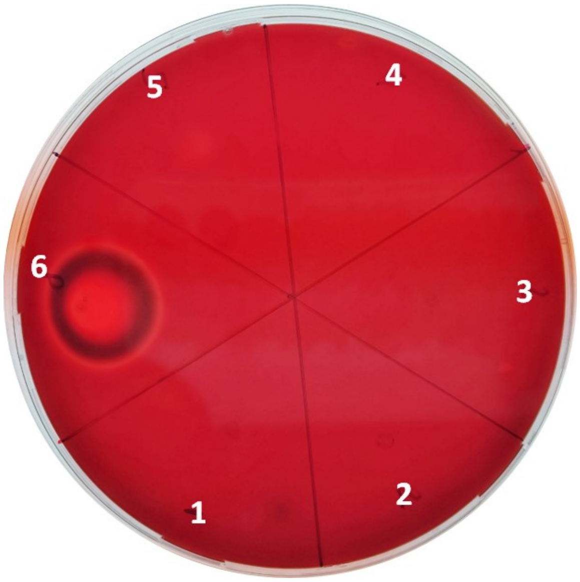

Figure S30. Representative image showing a horse blood agar plate with the indicated antimicrobial nanostructures. Nanoparticles (1:1 ratio of M1/M2:PY) were spotted on to horse blood agar plates and the plates were imaged post overnight incubation at 37 °C. A positive control was done with SDS (sodium dodecyl sulfate) to show hemolysis (white zone). 1-free PY, 2-spheriacl micelle, 3-nanoribbon, 4-short cylinders, 5-long cylinder, 6- Sodium dodecyl sulfate (SDS) surfactant, used as a positive control.

References:

1. Z. Li1, A. K. Pearce, J. Du, A. P. Dove and R. K. O'Reilly, *J. Polym. Sci.*, 2022,1.

2. F. Costanza, S. Padhee, H. Wu, Y. Wang, J. Revenis, C. Cao, Q. Li and J. Cai, *RSC Adv.,* 2014, **4**, 2089.

3. P. Chakraborty, H. K. Datta, P. Biswas and P. Dastidar, *Chem. Mater.,* 2021, **33**, 2274.

4. J. Bielawski, *Biochim Biophys Acta,* 1990, **1035**, 214.

5. M. Rekharsky, D. Hesek, M. Lee, S. O. Meroueh, Y. Inoue and S. Mobashery, *J. Am. Chem. Soc.*, 2006, **128**, 7736.
